# Uropathogenic *E. coli* Induce Different Immune Response in Testicular and Peritoneal Macrophages: Implications for Testicular Immune Privilege

**DOI:** 10.1371/journal.pone.0028452

**Published:** 2011-12-02

**Authors:** Sudhanshu Bhushan, Hamid Hossain, Yongning Lu, Andreas Geisler, Svetlin Tchatalbachev, Zbigniew Mikulski, Gerhard Schuler, Jörg Klug, Adrian Pilatz, Florian Wagenlehner, Trinad Chakraborty, Andreas Meinhardt

**Affiliations:** 1 Department of Anatomy and Cell Biology, Unit of Reproductive Biology, Justus-Liebig-University Giessen, Giessen, Germany; 2 Department of Medical Microbiology, Justus-Liebig-University Giessen, Giessen, Germany; 3 Clinic for Obstetrics, Gynecology and Andrology of Large and Small Animals, Justus-Liebig-University Giessen, Giessen, Germany; 4 Clinic of Urology, Pediatric Urology and Andrology, Justus-Liebig-University Giessen, Giessen, Germany; Charité, Campus Benjamin Franklin, Germany

## Abstract

Infertility affects one in seven couples and ascending bacterial infections of the male genitourinary tract by *Escherichia coli* are an important cause of male factor infertility. Thus understanding mechanisms by which immunocompetent cells such as testicular macrophages (TM) respond to infection and how bacterial pathogens manipulate defense pathways is of importance. Whole genome expression profiling of TM and peritoneal macrophages (PM) infected with uropathogenic *E. coli* (UPEC) revealed major differences in regulated genes. However, a multitude of genes implicated in calcium signaling pathways was a common feature which indicated a role of calcium-dependent nuclear factor of activated T cells (NFAT) signaling. UPEC-dependent NFAT activation was confirmed in both cultured TM and in TM in an *in vivo* UPEC infectious rat orchitis model. Elevated expression of NFATC2-regulated anti-inflammatory cytokines was found in TM (IL-4, IL-13) and PM (IL-3, IL-4, IL-13). NFATC2 is activated by rapid influx of calcium, an activity delineated to the pore forming toxin alpha-hemolysin by bacterial mutant analysis. Alpha-hemolysin suppressed IL-6 and TNF-α cytokine release from PM and caused differential activation of MAP kinase and AP-1 signaling pathways in TM and PM leading to reciprocal expression of key pro-inflammatory cytokines in PM (IL-1α, IL-1β, IL-6 downregulated) and TM (IL-1β, IL-6 upregulated). In addition, unlike PM, LPS-treated TM were refractory to NFκB activation shown by the absence of degradation of IκBα and lack of pro-inflammatory cytokine secretion (IL-6, TNF-α). Taken together, these results suggest a mechanism to the conundrum by which TM initiate immune responses to bacteria, while maintaining testicular immune privilege with its ability to tolerate neo-autoantigens expressed on developing spermatogenic cells.

## Introduction

Testicular macrophages (TM) represent the largest population of immune cells in the testis of mammals [1], [2]. They play an important role in the balance between defense against invading microorganisms and ‘testicular immune privilege’ which serves to protect the neo-antigens of the meiotic and haploid germ cells appearing during puberty after establishment of self-tolerance. Although TM exhibit many typical macrophage characteristics such as effective antigen presentation, phagocytotic functions as well as expression of Fc receptors and major histocompatibility complex (MHC) class II receptor [1], they are more reminiscent of a type-2 macrophage (M2) displaying diminished pro-inflammatory responses and reduced capacity to induce T cell activation [1], [3]–[6].

Male factor infertility is a common medical condition, affecting 1 in 20 men, with infections of the reproductive tract constituting the second most prevalent etiology either as primary cause or co-factor [7]–[10]. Bacterial infections of the testis are often derived from ascending urinary tract infections and frequently manifest as combined epididymo-orchitis caused predominantly by *Escherichia coli* or other *Enterobacteriaceae* pathogens [10]–[13]. Uropathogenic *E. coli* (UPEC) are known to be responsible for approximately 90% of urinary tract infections [14] and are frequently found in genital tract infections [15]. UPEC possess the ability to evade host defenses by blocking activation of NFκB, the archetypical transcription factor driving the expression of pro-inflammatory cytokines [16], [17]. Most of the genes encoding classical UPEC virulence factors are located on pathogenicity islands (PAIs). UPEC O6 strains 536 and CFT073 contain all major classes of virulence-associated factors including alpha-hemolysin [18]–[20]. UPEC alpha*-*hemolysin (HlyA) is a secreted pore forming toxin located on chromosomal PAIs. Integration of the toxin in the plasma membrane of host cells induces a rapid rise of cytoplasmic calcium levels through L-type calcium channels as well as from gated internal stores [21], an observation that stimulated by other agents is known to trigger downstream signaling cascades that lead to activation of important transcription factors including CREB, NFκB and NFAT [22], [23]. In a recent study, we showed that UPEC actively suppress MyD88-dependent Toll-like receptor signaling to prevent secretion of certain pro-inflammatory cytokines by testicular somatic cells including TM, a mechanism that facilitates pathogens survival and hence their detrimental actions on male fertility [16]. Although there is a general appreciation for the reduced capacity for pro-inflammatory gene expression in TM, there is a gap in our understanding as to how TM, on the one hand contribute to testicular pathogen recognition and defense signaling during bacterial infection, and on the other hand maintain immune privilege. Thus the objective of this study was (i) to elucidate the molecular details vital in this process set in comparison with another type of macrophage known to mount classical responses to pathogens such as peritoneal macrophages (PM) and (ii) to unravel UPEC virulence factors crucial in suppression of a pro-inflammatory immune response directed against the pathogen.

## Materials and Methods

### Animals

Adult male Wistar rats (249–270 g) were purchased from Harlan, Borchen, Germany and kept at 22°C with 14 h light:10 h dark schedule and fed with standard food pellets and water *ad libitum*. Animals were sacrificed by isofluran inhalation. This study was carried out in strict accordance with the recommendations in the Guide for the Care and Use of Laboratory Animals of the German law of animal welfare. The protocol was approved by the Committee on the ethics of Animal Experiments of the Regierungspraesidium Giessen, Giessen, Germany (permit number GI 20/23 –No. 16/2009). All surgery was performed under Ketamine and Xylazine anesthesia, and all efforts were made to minimize suffering.

### Antibodies and proteins

Antibodies directed against IκBα (#4814), phospho p38 (#9211), p38 (#9212), phospho ERK1/2 (#9106), ERK1/2 (#9102), phospho JNK1/2 (#9251), JNK1/2 (#9252) and phospho c-Jun (#3270) were all from Cell Signaling Technology. Wnt5a antibody was from Santa Cruz Biotechnology (Sc-30224), NFATC2 for Western blotting was from Abcam (Ab2722) and for immunofluorescence from Santa Cruz (Sc-13034), and the mouse monoclonal β-actin from Sigma (A5441). LPS (from *E. coli* 0127:B8) was purchased from Sigma. Recombinant IL-4 and IL-13 proteins were bought from Peprotech (Hamburg, Germany). HlyA was obtained from Prof. S. Bhakdi (Mainz University, Germany). HlyA was preincubated with polymyxin B (50 µg/ml) at 4°C for 30 min to remove any possible LPS contamination.

### Cell isolation

Testicular macrophages (TM) and peritoneal macrophages (PM) were isolated as described previously [16]. Purity of TM and PM was estimated 80–90% by double immunofluorescence staining using the rat macrophage specific antibodies directed against ED1 (CD68) and ED2 (CD163, Serotec, Oxford, UK, 1∶50 dilution. respectively).

### Bacterial strains

UPEC strain CFT073 (NCBI: AE014075, NC_004431) characterized by Welch, et al. [24], UPEC strain 536 (NCBI: NC_008253, CP000247 ), Brzuszkiewicz, et al. [20]. Pathogenicity islands deletion mutants isogenic to *E. coli* 536 were kindly provided by U. Dobrindt, University of Würzburg, Germany (Table 1). Non-pathogenic commensal *E. coli* (NPEC) strain 470, a human colon isolate (microbial collection of the Institute of Medical Microbiology, University of Giessen) and *E. coli* EPI300-T1^R^ (Epicentre Biotechnologies, Madison, Wisconsin) were used as controls. UPEC CFT073 (c2389::Kan) and UPEC 536 (EPC_1915::Kan) deletion mutants were constructed by the exchange of the TIR domain containing genes of each strain with a Kanamycin selection cassette using the lambda red system as described in Cirl, et al. [25]

**Table 1:** Genotype of UPEC strain 536 and deletion mutants.

Strain	Genotyp
*E. coli* 536	Wildtyp, Fim+, Sfa+, Pap+, Pix+, Hly I+, HlyII+, serum resistant, motile, Yersiniabactin+, Colibactin-
*E. coli* 536-114 (ΔPAI I)	PAI I−, Fim+, Sfa+, Pap+, HlyII+, serum resistant, motile, Yersiniabactin+, Colibactin-
*E. coli* 536-225 (ΔPAI II)	PAI II−, Fim−, Sfa+, Pap−, Pix+ HlyI+, serum sensitive, non motile, Yersiniabactin+, Colibactin−
*E. coli* 536 (ΔPAI III)	PAI III−, Fim+, Sfa−, Pap+, Pix+, Hly I+, HlyII+, serum resistant, motile, Yersiniabactin+, Colibactin−
*E. coli* 536ΔHPI (ΔPAI IV)	PAI IV−, Fim+, Sfa+, Pap+, Pix+ HlyII+, serum resistant, motile, Yersiniabactin−, Colibactin−
*E. coli* 536 (ΔPAI V)	PAI V−, Fim+, Sfa+, Pap+, Pix− HlyII+, serum resistant, motile, Yersiniabactin+, Colibactin−

### Construction of fosmid libraries

Fosmid libraries of UPEC strains *E. coli* 536 and CFT073 were constructed using the Copy Control Fosmid Library production kit (Epicenter Biotechnologies, Madison, Wisconsin) following the instructions of the manufacturer. Briefly, genomic DNA of each strain were sheared into fragments of>120 kb using ultrasound and end-repaired fragments were ligated into the pCC1FOS vector. Ligation reactions were coincubated with MaxPlax Lambda packaging extracts and used to transform EPI300-T1^R^ *E. coli*. Of each transformation 800 chloramphenicol resistant colonies were picked leading to an approximately six-fold coverage of the genome of *E. coli* 536 (4.94 Mb) and CFT073 (5.23 Mb), respectively. The resulting library clones were tested for hemolytic activity on blood agar plates. The inserts of the hemolysin positive library clones FOS 22 (CFT073) as well as clones FOS 2 and FOS 9 (*E. coli* 536) were sequenced. The insert in FOS 2 contained base pairs 396,6326 – 400,3202 of the *E. coli* 536 genome rendering it to the pathogenicity island (PAI) I and the *hlyA* gene hly II. FOS 9 contains base pairs 475,7325 – 479,9016 of the same genome which belong to the PAI II containing the second known *hlyA* gene of this strain hly II. FOS 22 spans base pairs 3400446–3433092 of the CFT073 genome enclosing the hemolysin *hlyA* gene.

### Propagation of bacteria and infection of cells

Bacterial strains were propagated over night on blood agar plates (Oxoid, Wesel, Germany). Fosmid carrying clones were propagated in LB medium, 20 µg/ml cloramphenicol. Fresh cultures were inoculated in LB medium and grown to early exponential phase (OD_600_ = 0.5–1.0) at 37°C in a shaking incubator. The concentration of viable bacteria was calculated using standard growth curves. Bacteria (2×10^9^) were centrifuged at 4,500 x g for 8 min at room temperature. The pellet was washed once at room temperature with PBS and taken up in 10 ml DMEM or RPMI 1640 medium (Invitrogen, Darmstadt, Germany). Serial dilutions in the same medium were performed and 100 µl were used to infect cells in one well of a 6-well cell culture plate (Sarstedt AG, Nuembrecht, Germany). For the recordings of intracellular calcium concentration ([Ca^2+^]_i_), bacteria were washed and resuspended in HEPES buffer (pH 7.4).

### Measurement of intracellular calcium concentrations

Recordings of intracellular calcium concentration ([Ca^2+^]_i_) were performed as described previously [26]. Measurements were performed in HEPES buffer containing 5.6 mM KCl, 141 mM NaCl, 1 mM MgCl_2_, 2.2 mM CaCl_2_, 11 mM D-glucose, 10 mM HEPES. Cells were loaded for 30 min with 3.3 µM Fura-2 AM (Invitrogen) and washed 3×10 min. Fluorescence images were taken with a slow scan CCD camera system with fast monochromator (TILL Photonics, Gräfelfing, Germany) coupled to an inverted microscope with a 20 x water immersion objective (Olympus, Hamburg, Germany). Fura-2 AM was excited at 340 and 380 nm wavelengths (λ), and fluorescence was collected at λ>420 nm. Cells were exposed to bacteria (UPEC CFT073, UPEC 536, NPEC 470 and UPEC 536 HDM diluted in HEPES buffer. Baseline recordings were performed with HEPES buffer only. Each cell was independently tracked and the fluorescence intensity ratio of 340/380 nm was recorded. Ratio values were normalized to 100% at the beginning of recording. Curves were plotted from recordings collected from cells isolated from three different animals for each experimental setup.

### Microarray Target Labeling and Hybridization

Testicular macrophages (TM, 3 groups) 1A: Control (not infected); 1B: TM + UPEC CFT073 30 Min; 1C: TM + UPEC CFT073 60 Min. Each sample was prepared in duplicate ( = 2 independent biological replicates per sample, total 6 samples). Peritoneal macrophages (PM, 3 groups) 2A: Control (not infected); 2B: PM + UPEC CFT073 30 Min; 2C: PM + UPEC CFT073 60 Min. Each sample was prepared in duplicate ( = 2 independent biological replicates per sample, total 6 samples).

Sample preparation was performed using the MessageAmp II Kit (Ambion, Applied Biosystems) following the manufacturer’s original protocol. Briefly, 1 µg total RNA were used in cDNA synthesis reactions with a poly-A binding primer containing the T7-polymerase promoter. Resulting cDNA was transcribed into cRNA in one round amplification in the presence of 11-Bio-UTP. Double stranded cDNA and biotin labeled cRNA were purified using the mini columns included in the kit. The eluted cRNA was quantified with a NanoDrop spectrophotometer (NanoDrop Technologies, Rockland DE, USA) and quality was assessed using the Agilent 2100 Bioanalyzer (Agilent Technologies). Portions of 20 µg cRNA were subjected to fragmentation in the presence of Mg^2+^. Subsequently, 10 µg fragmented cRNA (target) was loaded onto CodeLink Rat Whole Genome Microarray glass slides containing 35.129 probe sets (Applied Microarrays, Tempe AZ, USA) and hybridized for 18 h in a Minitron shaker incubator (Infors AG, Bottmingen, Germany) at 37°C/300 rpm. Washing and dyeing with Cy-5 coupled streptavidin followed the original protocol for CodeLink arrays (Applied Microarrays). Arrays were scanned using a GenePix 4000 B scanner and GenePix Pro 4.0 Software (Axon Instruments, Arlington, USA). Each RNA sample was hybridized onto two microarrays ( = 2 technical replicates). A total of 24 microarrays (4 per group) were subjected to data analyses.

### Microarray Data Analysis

Details of the microarray data analysis are described in Method S1. Complete data are available at the Gene Expression Omnibus (GEO) database (https://www.ncbi.nlm.nih.gov/geo/, accession number GSE24780). This study adhered to the MIAME standards [27].

### ELISA

TM and PM were treated with 10 µg/ml LPS, 20 ng/ml HlyA, 100 ng/ml IL-4, 100 ng/ml IL-13 and with bacteria (for details of bacterial strains see Table 1). Cell supernatants were collected at indicated time points in respective figures and assayed for TNF-α (e-bioscience, Frankfurt, Germany) and IL-6 (DuoSet, R&D Systems, Wiesbaden, Germany) by ELISA following the manufacturer’s instructions.

### Western blot

After treatment cells were lysed on ice for 30 min in RIPA buffer (50 mM Tris-HCl (pH 8), 150 mM NaCl, 1% Igepal CA-630 (v/v), 0.5% sodium deoxycholate, 0.1% SDS, 1 mM sodium orthovanadate, sodium fluoride 10 mM, 1 mM DTT and protease inhibitor mixture (Sigma-Aldrich)). Lysates were cleared by centrifugation (16,000 x *g* for 15 min at 4°C) and the protein concentration was determined by Bradford protein assay (Bio-Rad). Subsequently, 20-40 µg of protein were separated on 7–10% sodium dodecyl sulfate-polyacrylamide gel (SDS-PAGE) and blotted onto nitrocellulose membrane (Hybond-ECL (0.2 µm); GE Healthcare, Freiburg, Germany). Membranes were blocked with 5% nonfat dry milk for 1 h in TBS (20 mM Tris-HCl, pH 7.6, 150 mM NaCl) containing 0.1% Tween 20 (v/v) (TBS/Tween) and subsequently incubated with antibodies against NFATC2, wnt5a, p38, phospho-p38, ERK1/2, phospho-ERK1/2, JNK, phospho-JNK, phosphor-c-JUN and IκBα in 5% nonfat milk overnight at 4°C. Bands were visualized using ECL (GE Healthcare). Membranes were stripped and reprobed with an anti-actin antibody as loading control.

### In vivo orchitis model

Bacterial orchitis was elicited as described previously [16]. Seven days post UPEC infection testicular macrophages were isolated and stained using antibodies raised against NFATC2 and the macrophage markers ED1+ and ED2+.

### Immunofluorescence

TM and PM were treated with UPEC CFT073 (m.o.i. 20) and HlyA for 30 min, washed 3 times with phosphate buffer saline (PBS, pH 7.4) and then fixed with icecold 4% formaldehyde in PBS for 30 min at room temperature. After permeabilisation with 0.2% Triton X-100, unspecific protein binding was blocked by incubation for 1 h in PBS containing 5% normal goat serum and 5% BSA. Rabbit polyclonal anti-mouse NFATC2 antibody diluted 1∶50 in PBS containing 0.05% Tween 20 was added overnight at 4^°^C followed by incubation with Cy3-labeled secondary antibody for 1 h. Cell nuclei were counterstained with Cy5-labeled To-PRO-3 (Molecular Probes) and finally analyzed using a TCS SP2 confocal scanning microscope (Leica Microsystems, Wetzlar, Germany). For ED1 and ED2 staining in UPEC infected testis, cryosections (10 µm) were cut and fixed in 4% paraformaldehyde for 20 min. Unspecific protein binding was blocked as described above and samples were incubated with both primary antibodies (mouse anti-rat CD68 (ED1) and CD163 (ED2) 1:50; Serotec, Oxford, UK) at 4°C overnight followed by decoration with anti-mouse FITC-conjugated secondary antibody diluted in PBS for 1 h at RT in the dark. The slides were mounted with Vectashield Mounting Medium containing DAPI (Vector, Burlingame, USA).

### TUNEL assay

TUNEL assay was performed by using ApopTag® Fluorescein In Situ Apoptosis Detection Kit (Millipore, CA, USA) following the manufacture’s instructions. Percentage of TUNEL-positive TM and PM was determined at 400-fold magnification.

### Real time RT-PCR

Total RNA was isolated from UPEC CFT073 and HlyA treated TM and PM by using the RNeasy mini kit (Qiagen, Hilden, Germany). Contaminating DNA was removed by addition of 1 U DNase I (Invitrogen) per µg of total RNA and reverse transcription was performed for 1 h at 42°C in a 40 µl reaction using 200 U of moloney murine leukemia virus reverse transcriptase (Promega, Mannheim, Germany). Quantitative RT-PCR (qRT-PCR) was performed in an iCycler RT-PCR system (Bio-Rad, Munich, Germany) using the iQ^TM^ SYBR^®^ Green PCR kit (Bio-Rad, Munich, Germany). The PCR amplification condition for each primer set includes initial denaturation for one cycle (95°C for 8 min), 45 cycles of denaturation (95°C for 20 s), annealing (Table 2) and extension (72°C for 30 s). IL-3, IL-4, IL-10 and IL-13 primers were purchased from Qiagen (Hilden, Germany) and PCR amplification conditions were followed according to the manufacturer’s recommendations. The relative quantification of PCR products was determined by the comparative Ct method. The target gene expression was normalized by the non-regulated reference gene (β2 M) in UPEC treated samples and with β-actin in HlyA treated samples. Data were presented as relative expression (RE): RE = 2^ΔCtUPEC-ΔCt Ctrl^, ΔCt = Ct_target gene_-Ctβ_2 M/_β_-actin_.

**Table 2:**
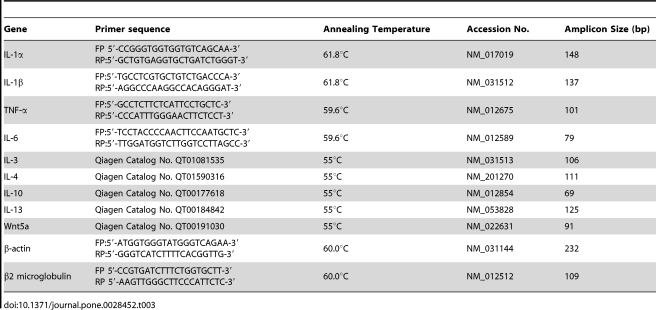
Sequences of forward (FP) and reverse primers (RP).

### Radioimmunoassay

Testosterone levels in testicular homogenates were measured after extraction of the samples with toluene by radioimmunoassay (RIA) as previously described [28]. The intra- and interassay coefficients of variation were 7.8% and 9%, respectively. The lower limit of detection was 0.1 ng/ml.

### Statistical analysis

Levels of mRNA or cytokine concentrations under different culture conditions were correlated to control cultures (normalized to 100%) and are represented as mean ± standard deviation (SD). Significance levels for ELISA were determined using Tukey-Kramer multiple comparisons test and Mann-Whitney U test for qRT-PCR data. For the statistical analysis of [Ca^2+^]_i_ measurements, area under the curve was calculated by summing up values obtained for each cell. Non-parametric rank based Kruskal-Wallis test was used to compare multiple groups and if significant differences were detected, it was followed by Mann-Whitney test to compare between two experimental groups. Differences in numbers of cells reacting to increasing concentrations of HlyA were measured by Fischer’s exact test. Tests were performed using SPSS software (SPSS software, Munich, Germany). P≤0.05 was considered significant and P≤0.01 as highly significant.

## Results

### UPEC and UPEC pathogenic island deletion mutants do not induce pro-inflammatory cytokine secretion

**Figure 1:**
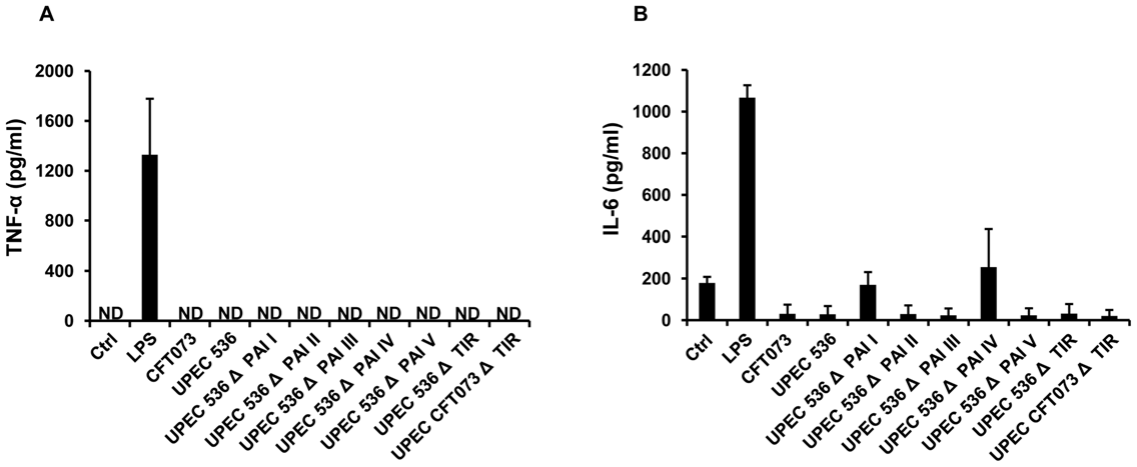
UPEC do not induce cytokine secretion in PM. PM were infected with UPEC CFT073, UPEC 536, pathogenic island mutants of UPEC 536 (ΔPAI I-V) as well as a TIR domain deletion mutant of UPEC CFT073 (UPEC CFT073 ΔTIR) and UPEC 536 (UPEC 536 ΔTIR) for 5 h (MOI = 0.1). Incubation with wild type UPEC strains (CFT073, 536) and their mutants did not cause secretion of (**A**) TNF-α or (**B**) IL-6 from cultured PM. As positive control PM were treated with 10 µg/ml LPS to induce IL-6 and TNF-α secretion. ND = not detectable. Significance levels for ELISA were determined using Tukey-Kramer multiple comparison test and p≤0.05 was considered significant.

Our previous data using peritoneal macrophages (PM) and other studies have demonstrated the ability of UPEC to suppress pro-inflammatory cytokine secretion in host cells [14], [16], [25]. TM showed no basal TNFα protein secretion or after challenge with LPS, NPEC and UPEC 536 (HDM) after 5 h (data not shown). TM are known to secrete pro-inflammatory cytokines after longer periods of LPS challenge [1], [3]–[6], but bacterial multiplication did not allow incubation times more than a few hours. Thus to determine which virulence factor of UPEC is responsible for inhibition of pro-inflammatory cytokine production, isolated rat PM were incubated with a set of isogenic deletion mutants lacking various PAIs and combinations thereof (Table 1). Infection with wild type UPEC CFT073 and 536 as well all five PAI deletion mutants failed to induce IL-6 or TNF-α secretion and even suppressed the production of IL-6 below basal levels. In contrast, LPS alone induced a strong cytokine secretion (Figure 1). Challenge of PM with the TIR deletion mutant of UPEC strain CFT073 (c2398::kan) and 536 (ECP_1915::kan) had no effect on TNF-α production and resulted in suppression of basal IL-6 secretion (Figure 1).

### Differential gene expression in testicular and peritoneal macrophages after UPEC infection

**Figure 2:**
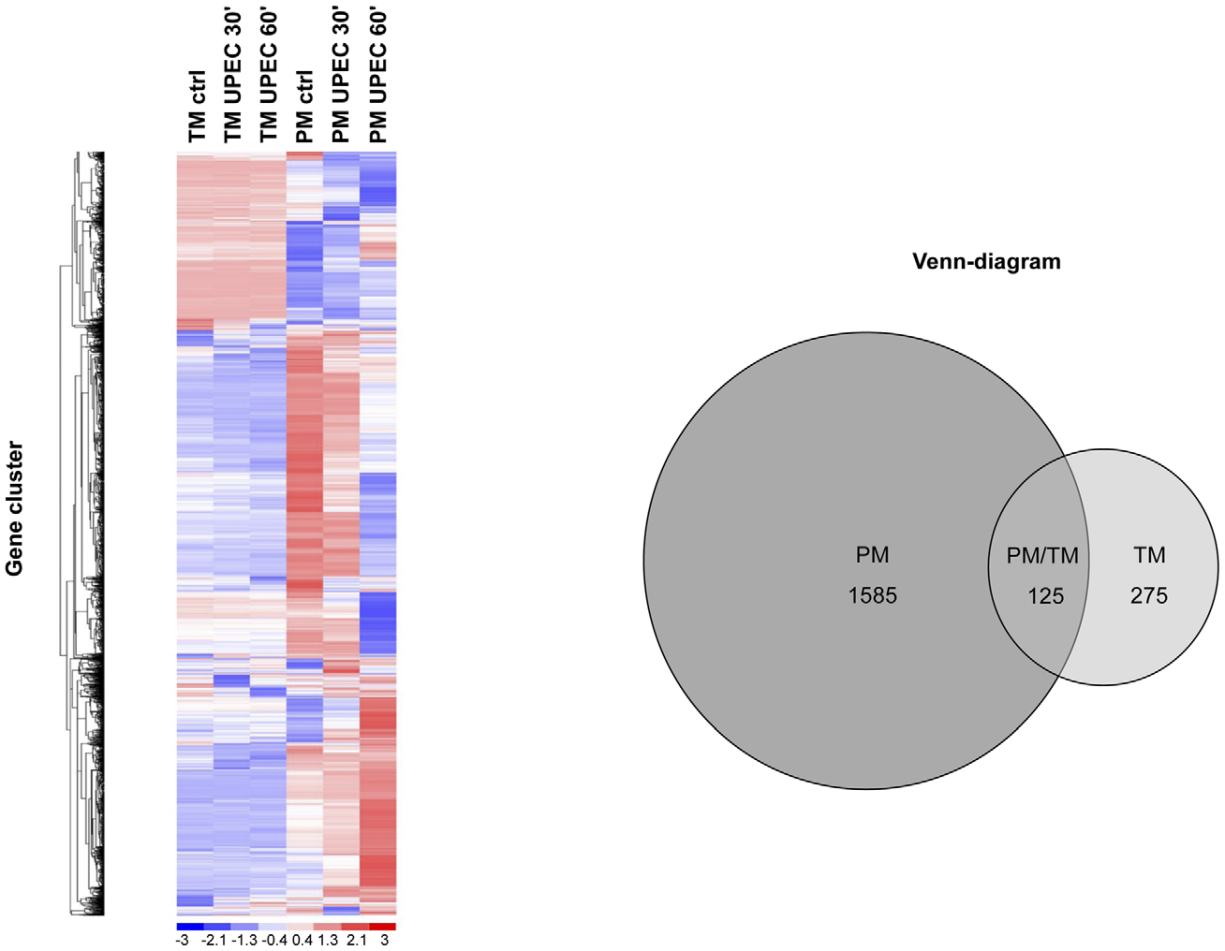
Hierarchical clustering of significantly regulated genes in PM and TM clearly distinguished between PM and TM pretreatment, 30 min and 60 min after infection with UPEC CFT073. (**A**) Blue indicates downregulation and red upregulation of genes. A time-dependent pattern of gene regulation can be observed within the macrophage populations. The picture clearly demonstrates how different PM and TM respond on the gene expression level upon infection with the same UPEC strain. (**B**) **Venn diagram of significantly regulated genes in PM and TM.** A total of 1710 genes (PM) and 400 genes (TM) were significantly regulated (FDR<0.05) at both time points 30 and 60 min. Both PM and TM showed an overlap of 125 genes with PM having 1585 unique genes and TM 275 unique genes.

As TM respond to UPEC infection with noticeable changes in mRNA expression after short periods of time [16], whole genome gene expression profiling of PM and TM was performed before and after infection with UPEC CFT073 to analyze both the molecular mechanisms responsible for the compromised pro-inflammatory function of TM and guide to unravel UPEC virulence factors with the ability to manipulate immune responses in both macrophage populations. PM responded with a much larger number of differentially regulated genes than TM (PM: 1710 genes, TM: 400 genes; total of both times points 30 min and 60 min, Table S1A-C). Only a small number of genes (n = 125) showed similar regulation in both macrophage populations (Figure 2, Table S1C). Hence, after infection with UPEC PM mobilized 5.3% of the genome, while TM mobilized only 1.2% of the genome (gene list available under the Gene Expression Omnibus (GEO) database (https://www.ncbi.nlm.nih.gov/geo/, accession number GSE24780)). Hierarchical clustering of the significantly regulated genes showed two clusters clearly differentiating between PM and TM (Figure 2). Gene ontology of the differentially regulated genes showed in PM early and sustained downregulation of genes involved in immune response (30 and 60 min) and delayed upregulation of genes involved in calcium homeostasis and anti-inflammation (60 min) (Table 3, Table S2A, S2B). On the other hand, the differentially regulated genes in TM showed an early but sustained upregulation of genes involved in both pro- and anti-inflammation and calcium homeostasis (Table 3, Table S2C, S2D). Interestingly, the data analysis revealed a multitude of genes implicated in calcium signaling pathways in both TM and PM highlighting the involvement of intracellular Ca^2+^ rises during infection with UPEC (Table S3). Therefore, we used the Ingenuity Pathways Analysis tool (Ingenuity® Systems) to identify genes/pathways centrally involved in cellular calcium signaling. We identified NFAT as a key molecule involved in calcium signaling as it is activated by Ca^2+^ via calcineurin and promotes transcription of the aforementioned inflammatory genes (Figure S1).

**Table 3:** Overrepresented biological categories of significantly regulated genes in PM and TM using DAVID.

Gene	Primer sequence	Annealing Temperature	Accession No.	Amplicon Size (bp)
IL-1α	FP 5′-CCGGGTGGTGGTGTCAGCAA-3′ RP:5′-GCTGTGAGGTGCTGATCTGGGT-3′	61.8°C	NM_017019	148
IL-1β	FP:5′-TGCCTCGTGCTGTCTGACCCA-3′ RP:5′-AGGCCCAAGGCCACAGGGAT-3′	61.8°C	NM_031512	137
TNF-α	FP:5′-GCCTCTTCTCATTCCTGCTC-3′RP:5′-CCCATTTGGGAACTTCTCCT-3′	59.6°C	NM_012675	101
IL-6	FP:5′-TCCTACCCCAACTTCCAATGCTC-3′ RP:5′-TTGGATGGTCTTGGTCCTTAGCC-3′	59.6°C	NM_012589	79
IL-3	Qiagen Catalog No. QT01081535	55°C	NM_031513	106
IL-4	Qiagen Catalog No. QT01590316	55°C	NM_201270	111
IL-10	Qiagen Catalog No. QT00177618	55°C	NM_012854	69
IL-13	Qiagen Catalog No. QT00184842	55°C	NM_053828	125
Wnt5a	Qiagen Catalog No. QT00191030	55°C	NM_022631	91
β-actin	FP:5′-ATGGTGGGTATGGGTCAGAA-3′ RP:5′-GGGTCATCTTTTCACGGTTG-3′	60.0°C	NM_031144	232
β2 microglobulin	FP 5’-CCGTGATCTTTCTGGTGCTT-3′ RP 5′-AAGTTGGGCTTCCCATTCTC-3′	60.0°C	NM_012512	109

PM 30 min UPEC					
Categories FC down	No of genes	p-value	Categories FC Up	No of genes	p-value
Regeneration	76	0.0030	Development	54	0.0086
Development	101	0.0081	Secretion	38	0.0328
Immune response	72	0.0101	Transport	8	0.0434
Taxis	15	0.0102			
Metabolism	24	0.0273			
Immune cell activation	87	0.0488			

PM 60 min UPEC					
Categories FC down	No of genes	p-value	Categories FC Up	No of genes	p-value
Response	173	1.1874E-16	Ca^2+^ ion homeostasis	52	0.0040
Activation of immune response	121	1.4083E-09	Signal transduction	149	0.0063
Response to stimuli	236	6.2956E-09	Cell-cell signaling	74	0.0191
Response to immunogenic stimulus	62	2.9268E-08	IL-4 type immunity	20	0.0413
Taxis	62	1.313E-07	MAP Kinase regulation	34	0.0470

TM 30 min UPEC					
Categories down	No of genes	p-value	Categories FC Up	No of genes	p-value
Immune response	28	0.0013	Ca^2+^ homeostasis	8	0.0036
Regeneration	18	0.0034	Inflammatory response	44	0.0075
Endocytosis	12	0.0156	Transcriptional regulation	44	0.0129
Development	44	0.0302			

TM 60 min UPEC					
Categories down	No of genes	p-value	Categories Up	No of genes	p-value
Development	56	0.0001	Locomotion	10	4.08E-06
Regeneration	34	0.0005	Defense response	13	5.90E-06
Ion hemostasis	37	0.0009	Regulation of gene expression	15	1.73E-05
Response to organic substance	20	0.0065	Regulatory process	45	7.30E-05
			Regulation of transcription	25	9.80E-05

Significantly regulated genes (FDR<0.05) were first assigned to gene ontology and then to functional biological categories. A p-value for the likelihood of the enrichment of biological processes was calculated using the Gene Ontology (GO) public database for the following experimental groups: PM after treatment with UPEC for 30 min or 60 min; TM after treatment with UPEC for 30 min or 60 min.

### UPEC strongly activate calcium mobilization in macrophages

**Figure 3:**
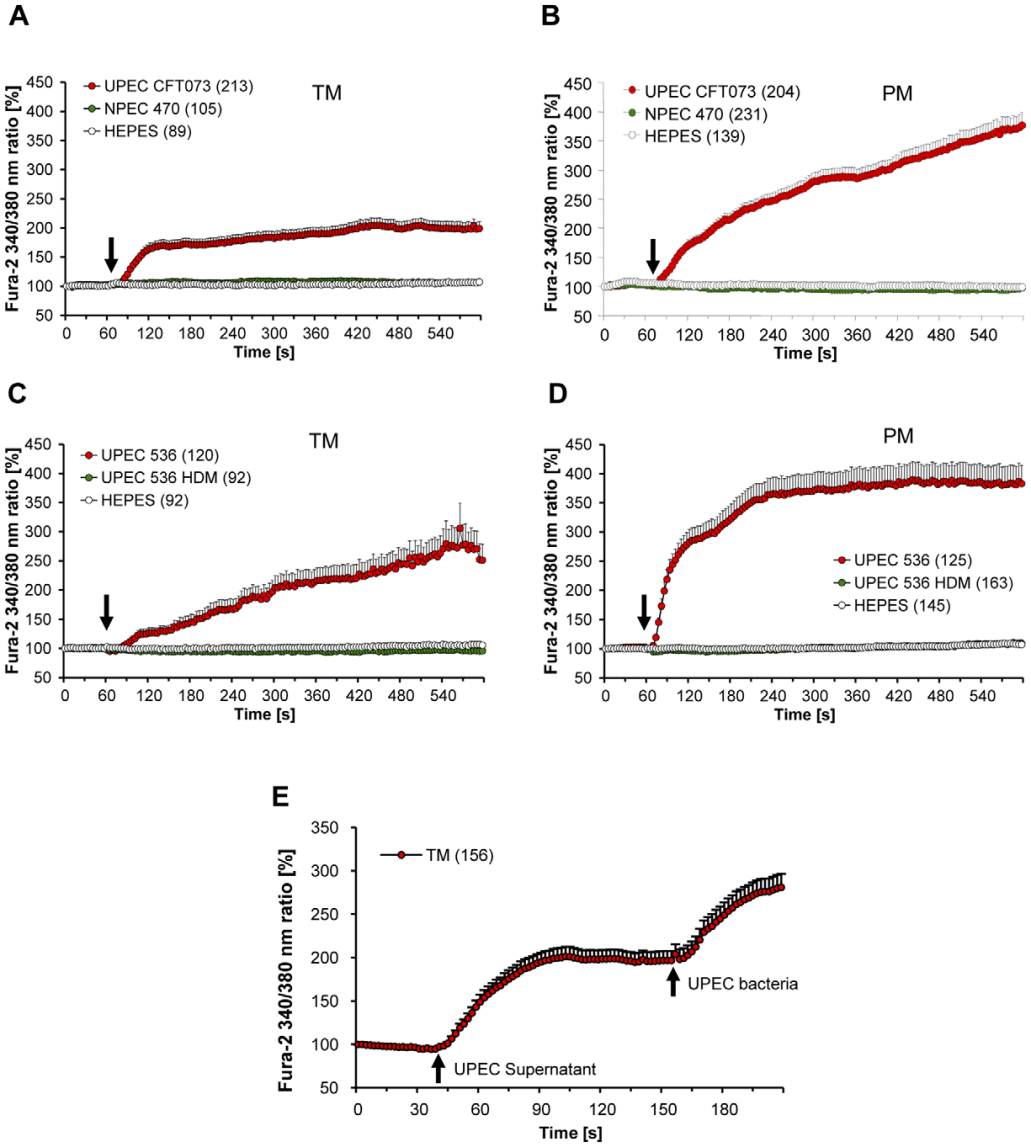
Ca^2+^ influx is dependent on presence of hemolysin genes in UPEC. UPEC strains CFT073 and 536 induced a Ca^2+^ influx in rat TM (**A, C**) and PM (**B, D**). After recording the baseline ratio of Fura-2 AM fluorescence after excitation at 340 nm versus excitation at 380 nm for 1 min, cells were treated with bacteria or vehicle as indicated by arrows. (**A, B**) PM and TM stimulated with UPEC CFT073 reveal a rapid and sustained increase in [Ca^2+^]_i_, while cells treated with vehicle (HEPES) or NPEC 470 do not respond with Ca^2+^ mobilization. (**C, D**) UPEC 536 deficient for HlyA (UPEC 536 HDM) were not effective in triggering a Ca^2+^ rise, whilst wildtype UPEC 536 elicited a rapid cytoplasmic Ca^2+^ influx in both PM and TM. Numbers of measured cells are given in brackets. (**E**) Soluble factor(s) present in the supernatants induced a rise in [Ca^2+^]_i_. UPEC bacteria were added as a positive control at the end of the experiment and caused further elevation of [Ca^2+^]_i_ levels. Intracellular Ca^2+^ was monitored by the Fura-2 method. Areas under the curve were calculated by summing up values obtained for each cell. Non-parametric rank based Kruskal-Wallis test was used to compare multiple groups and if significant differences were detected, it was followed by Mann-Whitney test to compare between two experimental groups.

To verify the effect of UPEC on calcium signaling, intracellular Ca^2+^ levels ([Ca^2+^]_I_ ) were recorded in PM and TM following infection. While commensal non-pathogenic *E. coli* (NPEC) strain NPEC 470 and vehicle control revealed no change on [Ca^2+^]_i_ treatment with UPEC CFT073 and 536 resulted in a rapid and profound rise in [Ca^2+^]_i_ (Figure 3). Similarly, supernatants of UPEC cultures were also effective in generating elevated [Ca^2+^]_I_ in TM suggesting the involvement of a secreted bacterial component (Figure 3). Applying increasing concentrations of purified UPEC hemolysin A (HlyA), a UPEC secreted pore forming toxin that is known to mediate rises of intracellular Ca^2+^ in target cells [21], resulted in a rapid dose-dependent increase of [Ca^2+^]_I_ in TM and PM. However, at lower concentrations of HlyA (1 and 5 ng/ml) a lower number of TM reacted than PM (Figure S3). Overall, PM consistently demonstrated a stronger increase in [Ca^2+^]_i_ compared to TM (p<0.001, Figure S3A). Moderate concentrations of purified HlyA (5 ng/ml) triggered well defined Ca^2+^ oscillations in TM (Figure S4). Likewise, UPEC 536 carrying two *hlyA* genes induced a stronger Ca^2+^ increase in both PM and TM than UPEC CFT073 which harbors only a single copy of the *hlyA* gene. Of note, deletion of both *hlyA* genes in the UPEC 536 strain (hlyA double mutant = UPEC 536 HDM) completely abolished the bacteria-induced rise in Ca^2+^ both in TM and PM to a level comparable to the wild type non-pathogenic NPEC 470 strain which does not produce HlyA (p<0.001, Figure 3).

### UPEC virulence factor HlyA suppresses pro-inflammatory cytokine secretion

**Figure 4:**
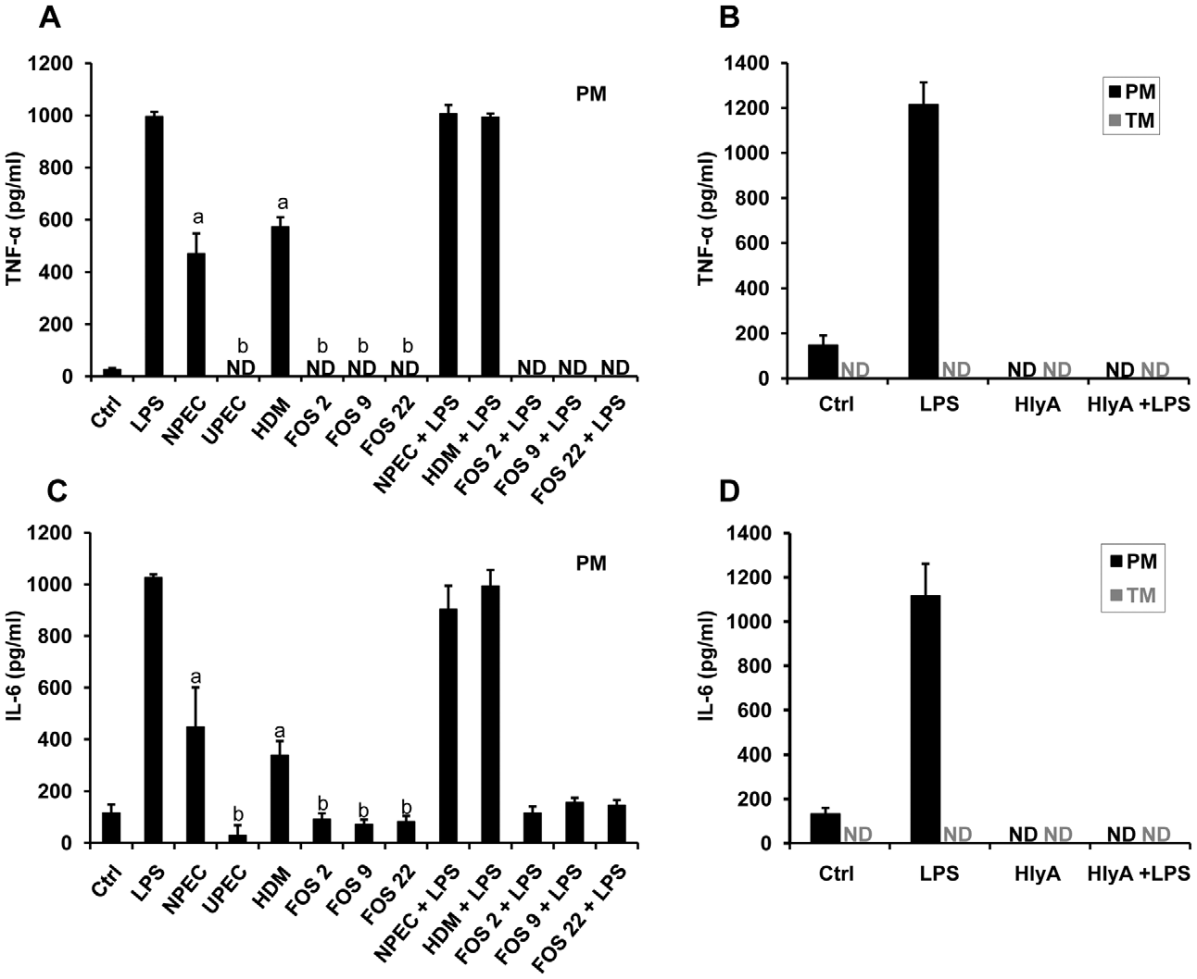
UPEC virulence factor hemolysin inhibits pro-inflammatory cytokine secretion. (**A, C**) PM were infected with NPEC mutants expressing hemolysin (FOS 2, FOS 9 and FOS 22), UPEC 536 hemolysin double mutant and wild type NPEC EPI-T1^R^ strain for 5 h with or without concomitant LPS stimulation. (**B, D**) PM and TM were treated with 20 ng/ml hemolysin in the presence of LPS or without for 5 h. IL-6 and TNF-α concentrations in culture supernatants were measured by sandwich ELISA. Values are means ± SD of triplicates. MOI = 0.1. Tukey-Kramer multiple comparisons test was used to analyze significance of data. Values with different letters superscript differ significantly (p<0.001) compared to NPEC and HDM. ND = not detectable.

To investigate a role of HlyA in the suppression of pro-inflammatory cytokine production, fosmid genomic libraries of UPEC 536 and CFT073 in the non-pathogenic K12 *E. coli* strain (EPI300-T1^R^) were generated and NPEC clones FOS 2, FOS 9 and FOS 22 ectopically expressing hemolysin were used for infection experiments. Of note, LPS-stimulated macrophages that were co-incubated with *E. coli* EPI300-T1^R^ strains ectopically expressing either of the two *hly*A genes of UPEC536 (pFOS2, pFOS9) or the single *hly*A gene of strain CFT073 (pFOS22) suppressed basal and LPS-induced IL-6 and TNF-α secretion from PM. This is in contrast to incubation with LPS and NPEC EPI300-T1^R^ which triggered the release of substantial amounts of IL-6 and TNF-α (Figure 4A, 4C). Deletion of both *hlyA* clusters in UPEC 536 (HDM) reversed the suppression of TNF-α and IL-6 secretion to a level comparable to that of NPEC. Similarly to NPEC expressing ectopically HlyA, purified HlyA also abolished basal and LPS-stimulated TNF-α and IL-6 release in PM (Figure 4B, 4D).

### UPEC alpha-hemolysin activates NFAT signaling pathways

**Figure 5:**
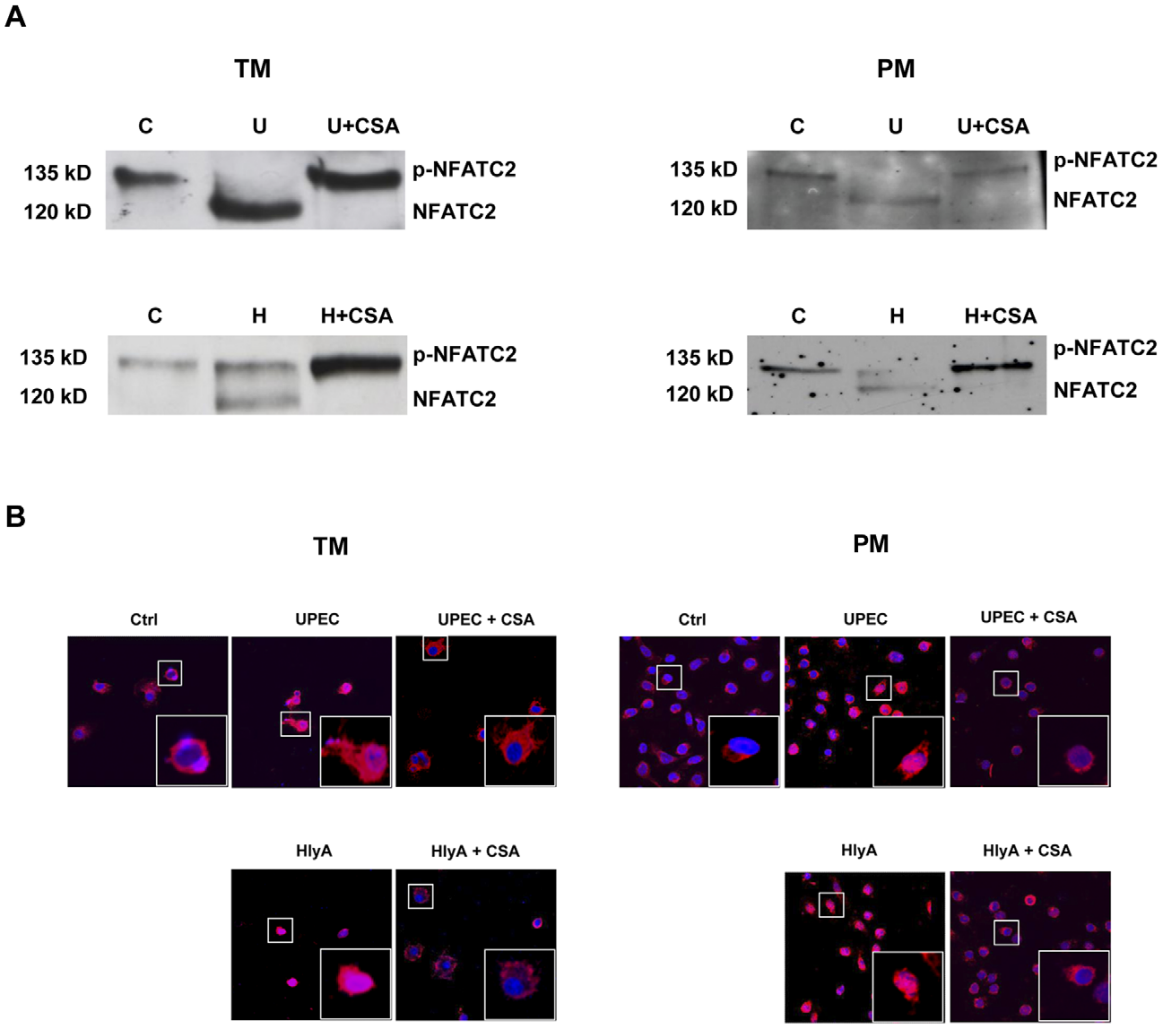
UPEC activates NFATC2 signaling pathway. (**A, B**) PM and TM were treated with UPEC CFT073 (MOI = 20) for 1 h. (**A**) TM and PM were pretreated for 15 min with 2 µM cyclosporine A (CSA) or left unstimulated, followed by incubation with UPEC CFT073 or 40 ng/ml alpha-hemolysin (H) for 30 min, respectively. For Western blot analysis 40 µg of protein were separated on a 7.5% SDS-PAGE and immunoblots were probed using an anti-NFATC2 antibody (NFAT). (**B**) UPEC CFT073 and alpha-hemolysin (HlyA) induced calcineurin-dependent NFATC2 nuclear translocation. TM and PM were pretreated with 2 µM of the calcineurin inhibitor CSA for 15 min or left unstimulated with subsequent challenge by UPEC (MOI = 20) or 40 ng/ml HlyA for 30 min. In support of the data shown in (**B**), nuclear translocation of NFATC2 (red) after UPEC CFT073 and HlyA treatment was observed in both TM and PM. Pretreatment of PM and TM with CSA blocked NFATC2 nuclear translocation. Nuclei were counterstained with Cy5-conjugated TO-PRO-3.

Incubation with UPEC CFT073 or purified HlyA resulted in a Ca^2+^/calmodulin and calcineurin dependent activation of NFATC2 in TM and PM (Figure 5A). Calcineurin-dependent dephosphorylation and subsequent mobility shift of the phosphorylated form (ca. 135 kD) to the unphosphorylated form of NFATC2 (ca. 120 kD) was visualized by Western blot analysis and confirmed by using the calcineurin antagonist cyclosporin A (Figure 5A). Dephosphorylation of NFATC2 leads to its translocation to the nucleus. Indeed, following addition of UPEC or purified HlyA for 30 min nuclear translocation of cytosolic NFATC2 is observed in both TM and PM, a process that is effectively inhibited by cyclosporin A (Figure 5B).

### UPEC activates transcription of anti-inflammatory cytokine genes in TM and PM

**Figure 6:**
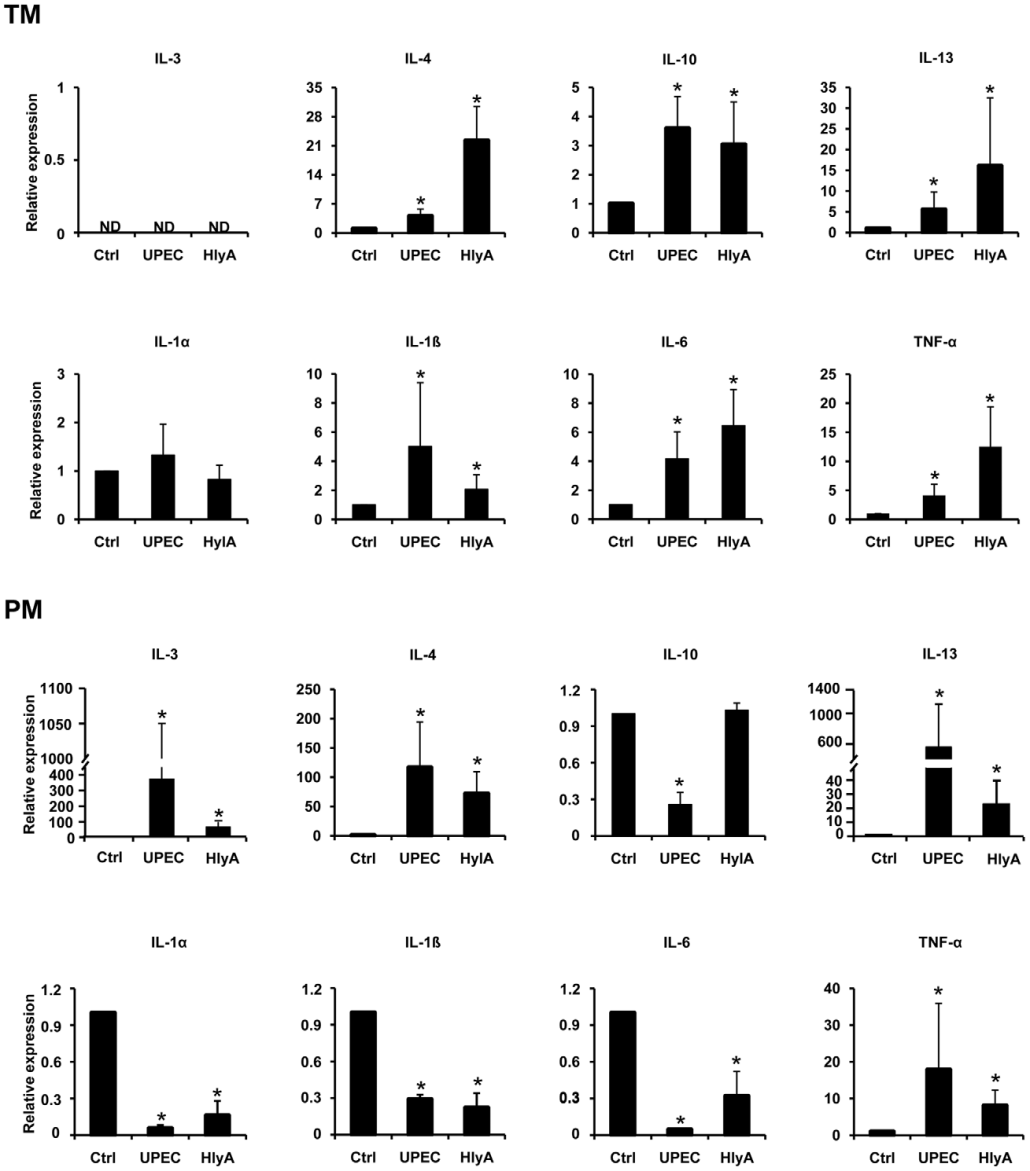
qRT-PCR analyses of anti-inflammatory cytokines (IL-3, IL-4, IL-10, IL-13, upper row) and pro-inflammatory cytokines (IL1-α, IL1-β, IL-6, TNF-α, lower row) after challenge of TM and PM with UPEC CFT073 (MOI = 20) or hemolysin A (HlyA, 40 ng/ml) for 1 h. Results were normalized using β-microglobulin and β-actin as endogenous controls and are shown as fold changes relative to uninfected controls. HlyA was preincubated with polymyxin B (50 µg/ml) at 4°C for 30 min to remove any possible LPS contamination. Values are means ± SD of triplicates. Mann-Whitney U test was used to analyze data and p≤0.05 was considered significant.

As NFATC2 is a major regulator of immunoregulatory and anti-inflammatory cytokines, expression of IL-3, IL-4, IL-10, and IL-13 which are known to be regulated by NFATC2 was examined by qRT-PCR (Figure 6A, 6B). Increased expression of the anti-inflammatory cytokines IL-4 and IL-13 was observed in both PM and TM after treatment with purified HlyA and UPEC CFT073 (Figure 6). Interestingly, IL-3 mRNA levels were undetectable in TM whilst expression increased approx. 300-fold in PM. Moreover, increased IL-10 mRNA levels were found in TM, whereas mRNA levels decreased (UPEC) or remained the same (HlyA) in treated PM (Figure 6A, 6B). Of note, expression of pro-inflammatory cytokines showed a differential response in TM and PM. In TM IL-1β, IL-6 and TNF-α mRNA levels are all elevated, only TNF-α shows the same response in PM, while IL-1α, IL-1β and IL-6 are downregulated by UPEC and HlyA (Figure 6). The involvement of NFATC2 in elevated expression of IL-4 and IL-13 following UPEC infection in TM and PM was verified using the NFAT pathway inhibitor cyclosporine A, which could suppress upregulated levels of these anti-inflammatory cytokines (Figure S5).

### HlyA induces differential response on MAP kinase activation and apoptosis ratio in PM and TM

**Figure 7:**
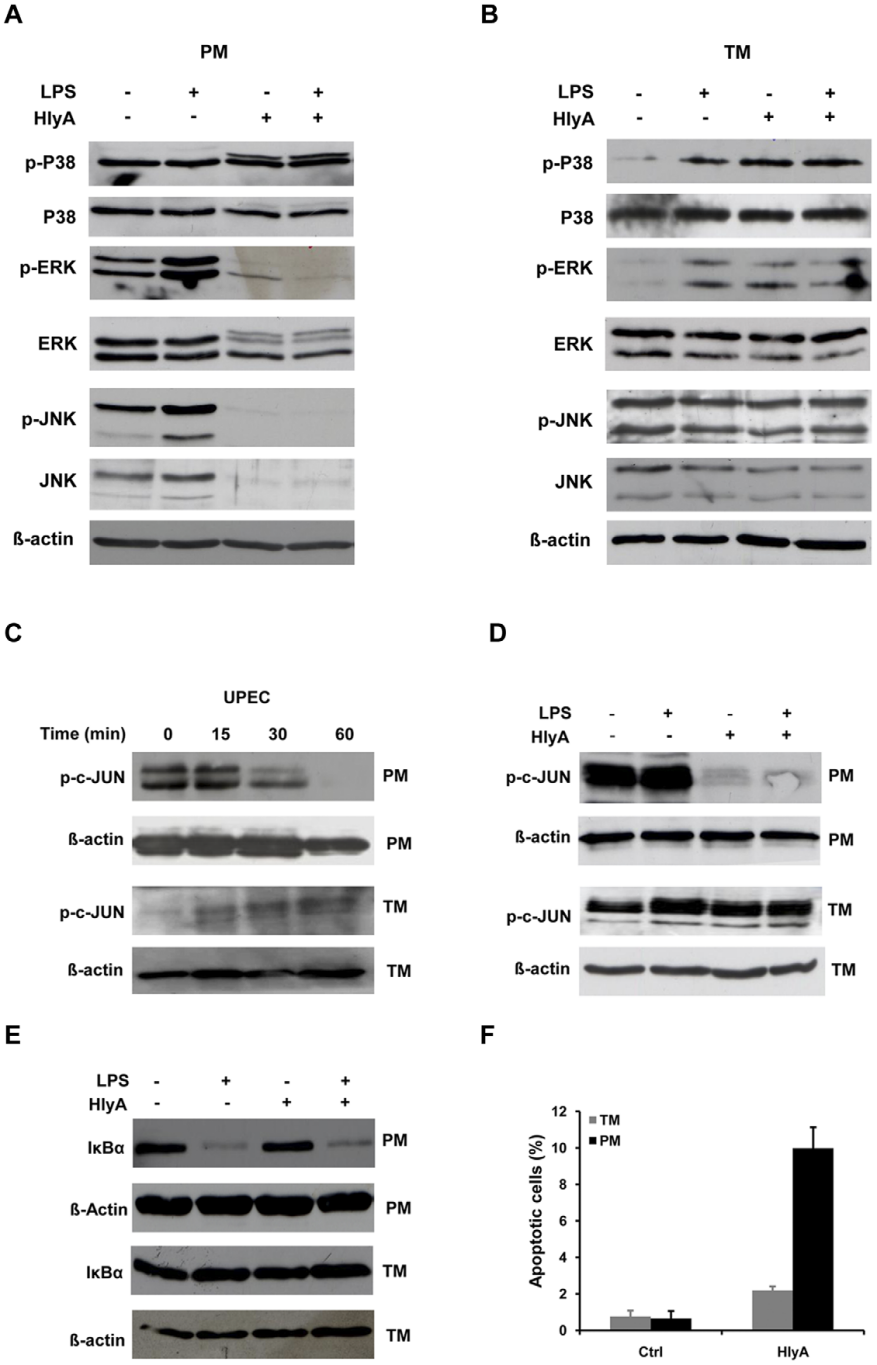
HlyA suppresses MAP kinase activation. (**A**) PM and (**B**) TM were treated with 10 µg/ml LPS and 40 ng/ml of alpha-hemolysin (HlyA) for 30 min as indicated. For Western blot analysis 20 µg of protein were separated by 10% SDS-PAGE and immunoblots were probed with antibodies specific for the phosphorylated forms of MAP kinases p38 (p-P38), JNK (p-JNK) and ERK 1/2 (p-ERK1/2), respectively. Changes in the total amount of kinases following treatment and equal loading of samples were assessed by detecting total levels of p38, JNK, and ERK1/2. β-actin levels served as general loading control. AP-1 signaling pathway activation in TM and PM was verified by assessing phospho-c-JUN (p-c-JUN) levels after treatment with (**C**) UPEC CFT073 (MOI = 20) for 30 min. (**D**) HlyA treatment strongly reduces p-c-JUN levels in LPS stimulated and unstimulated PM, but not in TM. (**E**) HlyA caused IκBα degradation in PM and TM. Cells were treated with LPS and/or HlyA for 30 min as indicated above and blots were probed with an anti-IκBα antibody. (**C-E**) β-actin detection served as loading control. Membranes in A-D were stripped and reprobed to test for the phosphorylated or unphosphorylated form or loading control, respectively. Antibodies against p-ERK and ERK were probed on split samples run on parallel gels. (**F**) HlyA induces apoptosis in PM, but not in TM. TM and PM were incubated with HlyA (40 ng/ml) for 1 h and DNA fragmentation was examined by the TUNEL assay as an index for apoptosis. HlyA was preincubated with polymyxin B (50 µg/ml) at 4°C for 30 min to remove any possible LPS contamination.

Based on our earlier observation of the effects of UPEC on MAP kinases in TM and PM [16], we investigated the role of HlyA in this process. Thus TM and PM were treated with purified HlyA for 30 min in the presence or absence of LPS. LPS induced phosphorylation of MAP kinases in TM (p38, ERK1/2) and PM (JNK, ERK1/2). Interestingly, purified HlyA strongly downregulated total protein expression of MAP kinases in unstimulated PM (JNK, ERK1/2) and in addition decreases phosphorylation in LPS-treated PM (JNK, ERK1/2). In contrast to PM, HlyA activates MAP kinases in TM (p38, ERK1/2) and showed no downregulation of either total MAP kinase levels or phosphorylation (Figure 7A, 7B). As MAP kinases are upstream factors in AP-1 signaling, we sought to determine whether HlyA also affects AP-1. In PM purified HlyA clearly attenuates phospho-c-Jun levels (Figure 7C). In contrast, in TM HlyA caused phosphorylation of c-Jun similar to the MAP kinase activation (Figure 7C).

Based on the observation that UPEC suppresses NFκB signaling pathway by stabilizing IκBα [17], we have examined the influence of HlyA on the stability of IκBα. HlyA showed no influence on IκBα levels in unstimulated and LPS challenged PM and TM (Figure 7E). In contrast to PM, IκBα did not degrade in TM after treatment with LPS (Figure 7E). The suppression of MAP kinases and AP-1 signaling pathways is known to induces apoptosis [29], [30], a fact verified in our study also for PM by using the JNK inhibitor SP600125 (Figure S7). As seen by TUNEL assay incubation with isolated HlyA for 1 h resulted in an increased ratio of apoptotic cells in both macrophage populations with PM (almost 10%) being much stronger affected than TM (<2%, Figure 7F).

### UPEC infection causes nuclear translocation of NFATC2 in TM in vivo

**Figure 8:**
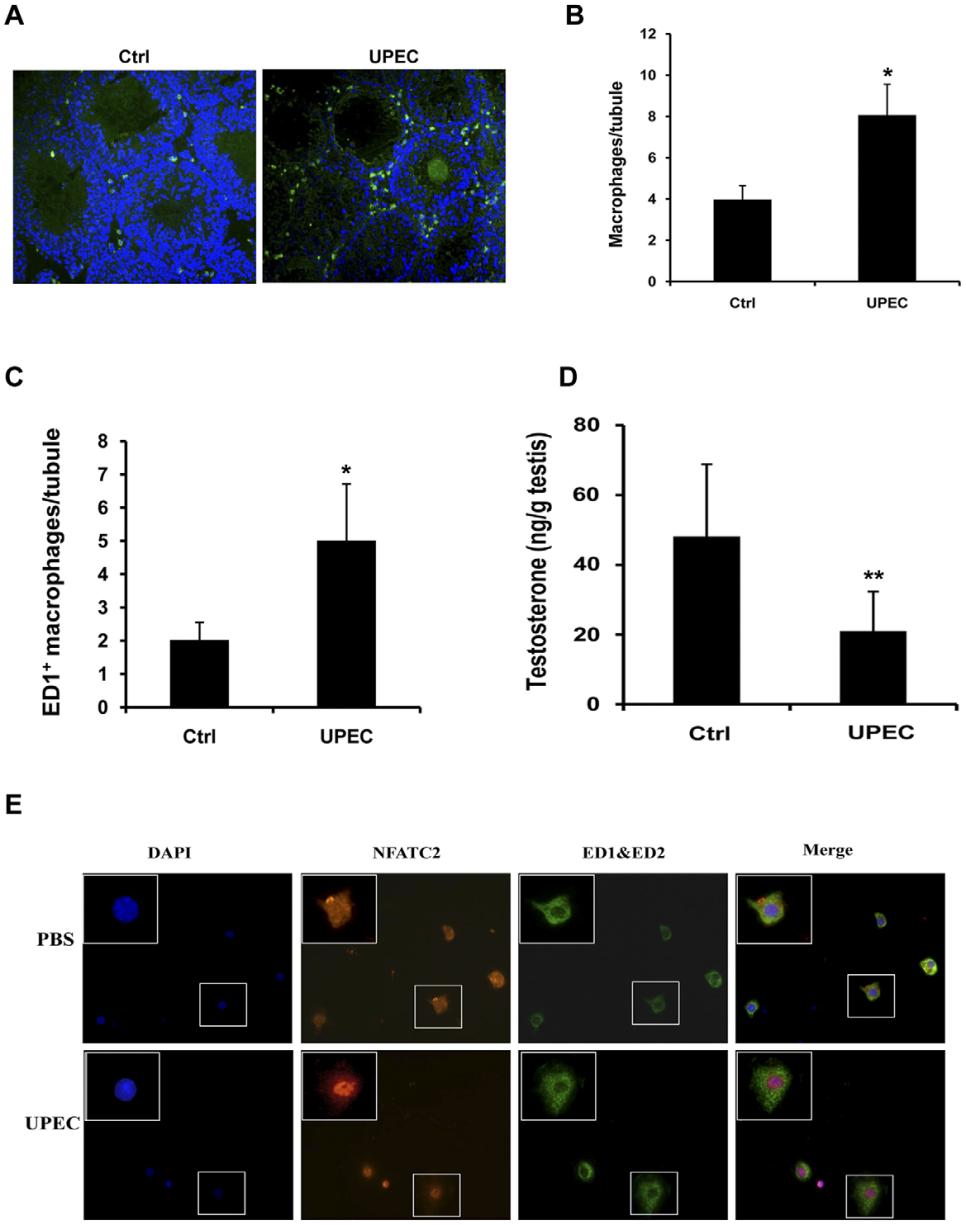
Bacterial orchitis elicited by *in vivo* infection with UPEC CFT073. Infection resulted in an increased number of (**A, B**) ED1+ED2+ total testicular macrophages and (**C**) ED1+ ‘inflammatory’ macrophages. Immunolabeled macrophages were counted in whole cross-sections and related to the number of cross-sectioned seminiferous tubules in the same section visualized by DAPI stain. (**D**) Testosterone concentrations were measured by RIA in testicular homogenates of testis obtained from animals 7 days post UPEC infection into the vas deferens and PBS injected sham operated rats (Ctrl). Testosterone concentrations are significantly lower in orchitis compared to sham-operated animals. (**E**) In TM isolated from testis 7 days post UPEC infection in the vas deferens, NFATC2 showed a nuclear localization. In contrast, NFATC2 is found in the cytoplasm of TM obtained from testis of PBS injected sham operated rats (Ctrl). Nuclei are counterstained with DAPI (blue). Inserts show a higher magnification of a cell seen in the respective overview. Macrophage quantification: values are means ± SD of n =  2 (Ctrl), n = 4 (UPEC). Testosterone measurements: values are means ± SD of Ctrl (n = 8); UPEC (n = 10). Statistical analysis was performed using student t-test. * = p< 0.05, ** = p<0.01.

Using an *in vivo* induced bacterial orchitis model, the total number of TM (ED1+/ED2+) in orchitis is significantly increased seven days post-UPEC infection compared to sham operated rats, a fact that can be at least partly attributed to the recruitment of ED1+ ‘newly arrived inflammatory’ macrophages which are found increased as well (Figure 8A-C). Intratesticular testosterone concentrations were found significantly lower in seven days post infection testes than in PBS-treated control testes (Figure 8D). Furthermore, using immunofluorescence ED1+/ED2+ TM isolated from PBS injected testes showed a cytoplasmic localization of NFATC2, whereas NFATC2 is found in the nucleus in TM obtained from UPEC infected inflamed testis seven days post infection (Figure 8E).

## Discussion

Inflammation of the testis as a complication of acute epididymitis due to ascending, canalicular bacterial infections is common and may occur in up to 60% of affected patients [7], [10]. Chronic inflammatory conditions of the testes can persist even after successful antibiotic therapy and can irreversibly alter sperm number and quality [31]. It is therefore relevant to understand the mechanisms by which immunocompetent cells such as the TM recognize and respond to infectious agents and how bacterial pathogens try to manipulate host defense in the testis.

Upon infection with UPEC, whole-genome based transcriptional analysis of host cell responses revealed highly distinct sets of regulated genes in TM and PM. Of note, a much narrower response of TM was seen that mobilized only 1.2% of the total genome in relation to PM (5.3%). Interestingly, gene ontology documented a sustained upregulation of genes implicated in both anti- and pro-inflammatory response in TM, which was not visible in PM, a fact that could reflect the need for a more delicately balanced and fine tuned immune response at the interface between testicular immune privilege and anti-bacterial response. Strikingly, in both macrophage populations genes involved in calcium homeostasis and signaling were commonly regulated. Thus, gene expression data suggest the involvement of a UPEC factor that mediates changes in intracellular calcium levels. In this study, we have shown that UPEC induced a rapid rise in [Ca^2+^]_i_ which is much more distinct in PM than in TM. Elevated intracellular Ca^2+^ levels in both TM and PM were found only after infection with UPEC, whereas NPEC or UPEC mutants devoid of *hlyA* genes elicited no response. This clearly pointed to an involvement of the pore forming virulence factor HlyA in altering calcium levels, an assumption confirmed by using purified HlyA in TM and PM.

An important signaling pathway in immune cells activated in response to calcium involves NFAT [22], [23], [32]. Specifically, NFAT activation requires sustained calcium levels as it was observed also with macrophages challenged with UPEC and HlyA in this study. Concomitantly, these treatments led to activation of NFATC2 in both TM and PM as documented by dephosphorylation and subsequent nuclear translocation of this transcription factor. Importantly, using an *in vivo* UPEC infection model, TM isolated from infected testes also revealed NFATC2 nuclear translocation confirming the *in vitro* observations. Blocking the Ca^2+^/calmodulin-dependent serine phosphatase calcineurin with cyclosporin A prevented dephosphorylation and nuclear import of NFATC2. To drive expression of IL-2, IL-3, IL-4, IL-5, IL-13, GM-CSF, IFN-γ and TNF-α a coordinated activation of NFAT family members together with AP-1 transcription factors is necessary [33]. Depending on whether or not both transcription factors are concomitantly activated, transcriptional activity results in the expression of two distinct gene sets, eliciting different patterns of cellular response. In contrast to the cytokines mentioned above, mutagenesis studies revealed that TNF-α and IL-13 promoter activity is independent of cooperative recruitment of AP-1 [34]. In our study, mRNA expression of pro-inflammatory cytokines IL-1α, IL-1β and IL-6 in PM, all of which require AP-1 for activation, were downregulated, while increased expression of IL-3, IL-4, IL-13 and TNF-α was observed. Thus our results using the NFAT signaling pathway inhibitor cyclosporine provide evidence that HlyA induced cytokine expression in PM is dependent on NFATC2 and independent of coordinated NFAT/AP-1 activity (Figure S5 and S6), whilst both NFAT and AP-1 signaling is activated in TM indicating their involvement in cytokine expression. A number of previous studies have demonstrated that UPEC can actively subvert immune responses by suppressing NFκB signaling [16], [17], [25], whilst a putative co-repression of AP-1 signaling by UPEC has received no attention yet. Our data indicate that AP-1 regulated transcription requires the activation of MAP kinases upstream of AP-1. In TM, purified HlyA activates MAP kinases and subsequent AP-1 dependent transcription as shown by increased phosphorylation of p38 and ERK1/2 kinases and synthesis of pro-inflammatory cytokines.

In contrast, downregulation of IL-1α, IL-1β and IL-6 mRNA expression in PM is thought to be related to a HlyA-dependent specific impairment of AP-1 signaling as seen by strong attenuation of total and phosphorylated MAP kinases as well as p-c-Jun levels. Furthermore, in confirmation with previous reports [29], [30] our results indicate that suppression of host MAP kinase signaling cascades by HlyA can lead to reduced viability and ultimately cell death and apoptosis in PM as suggested by use of a JNK inhibitor (Figure S7). Concomitant with a stimulation rather than inhibition of MAP kinase signaling, HlyA does not cause apoptosis in TM. In agreement, in our *in vivo* UPEC infection model, TM numbers are elevated seven days post-infection (Figure 8) and TM were almost exclusively negative using TUNEL staining (data not shown). This suggests that HlyA can subvert the outcome of an immune response in TM by modulating cytokine expression rather than killing cells. It is likely that their inflammatory products such as IL-1 and TNF-α are at least partly responsible for the decreased intratesticular testosterone concentrations found in infected testis due to direct inhibitory effects on androgen producing Leydig cells [1], [35], [36]. Alternatively/additionally TM are known producers of 25-hydroxycholestrol, an oxysterol that can negatively impact luteinizing hormone stimulated Leydig cell testosterone production [37]. Vice versa testosterone suppresses production of 25-hydroxycholestrol in TM pointing to an interesting control loop between TM and Leydig cells involving two factors with established potent inhibitory function on innate immune responses and likely candidates in establishment testicular immune privilege [38–40]. A further layer of complexity is added as TM secreted 25-hydroxycholestrol can be converted to testosterone in Leydig cells. As TM isolates can be contaminated by Leydig cells (usually 5–10%) an influence of both testosterone and oxysterols in the observed HlyA effects on TM cannot be fully excluded. However, under the experimental *in vitro* conditions used in this study the effect could at best be minor as both mediators are negatively regulating each other thus minimizing their impact on immune responses.

In summation, although the immune response of TM compared to PM is reduced by blockage of NFκB activation, cells maintain a general responsiveness by activation of MAP kinase and AP-1 signaling pathways following LPS and HlyA stimulation. These features enable TM to fulfill apparently paradox tasks, i.e. protection against microbes, while at the same time maintaining the immune privileged status of the testis to protect the developing germ cells.

## Supporting Information

Figure S1 **Calcium signaling pathway. (A)** Significantly regulated genes in PM and TM were used to generate calcium signaling pathways and identify genes within these pathways using Ingenuity Pathways Analysis (Ingenuity® Systems). Intracellular calcium signaling is mediated in PM 60 min after UPEC infection via calcineurin and nuclear translocation of NFAT which finally leads to the expression of inflammatory cytokines. Rises in intracellular calcium levels also activates protein kinase c (PKC) which in turn activates kinases such as MAPK and JNK. Up regulated genes are depicted in red. **B)** The picture demonstrates how UPEC alpha hemolysin (HlyA) increases intracellular calcium concentrations which then leads to translocation of NFAT after dephosphorylation by calcineurin.
(TIF)

Figure S2 **Area under curve analysis for [Ca^2+^]_I_ data set in** **Figure 3**. Data were calculated by summing up values obtained for each cell. Rise in [Ca^2+^]_I_ is caused in **(A, C)** TM and **(B, D)** PM by UPEC CFT073 and 536 strain, but not by NPEC 570 and UPEC 536 HDM which lack HlyA. A stronger Ca^2+^ influx in PM is clearly visible. Non-parametric rank based Kruskal-Wallis test was used to compare multiple groups and if significant differences were detected, it was followed by Mann-Whitney test to compare between two experimental groups.
(TIF)

Figure S3 **Effect of hemolysin challenge on Ca^2+^ influx in TM and PM**. **(A)** Cumulative dose-response curve. **(B)** Number of cells reacting to increasing concentrations of hemolysin A is shown. *** - P≤0.001; differences in numbers of cells reacting to increasing concentrations of HlyA were measured by Fischer’s exact test.
(TIF)

Figure S4 **Alpha-hemolysin caused intracellular Ca2+ oscillations in testicular macrophages (TM).** Cells were treated with purified alpha-hemolysin (5 ng/ml, as indicated by arrows) and [Ca2+]i was monitored for 30 min using the Fura-2 method.
(TIF)

Figure S5 **NFAT pathway inhibitor cyclosporine suppressed UPEC induced NFATC2 dependent expression of anti-inflammatory cytokine IL-4 and IL-13.** TM and PM were pretreated with 2 µM cyclosporine A (CSA) for 15 min prior challenge with UPEC (MOI = 20) for 1 h. Expression levels of IL-4 and IL-13 were analyzed using qRT-PCR. Results were normalized using β-microglobulin as endogenous controls and are shown as fold changes relative to uninfected controls. Values are means ± SD of triplicates. Mann-Whitney U test was used to analyze data. Values with different letters superscript differ significantly compared to control (Ctrl).
(TIF)

Figure S6 **NFAT pathway inhibitor cyclosporine suppressed UPEC induced NFATC2 dependent expression of TNF-**α **in PM.** PM were pretreated with 2 µM cyclosporine A (CSA) for 15 min prior challenge with UPEC (MOI = 20) for 1 h. Expression levels of TNF-α were analyzed using qRT-PCR. Results were normalized using β-microglobulin as endogenous controls and are shown as fold changes relative to uninfected controls. Values are means ± SD of triplicates. Mann-Whitney U test was used to analyze data. Values with different letters superscript differ significantly compared to control (Ctrl).
(TIF)

Figure S7 **The MAP kinase JNK inhibitor SP600125 induces cell death in PM.** **(A)** PM were treated with JNK inhibitor SP600125 at the indicated concentration for 24 h. Cell viability was determined using the colorimetric MTT assay. Results are presented as means ± SD of triplicates. Mann-Whitney U test was used to analyze data. Values with different letters superscript differ significantly compared to control (Ctrl). **(B)** PM were treated with 25 µM SP600125 JNK inhibitor for 24 h and DNA fragmentation was examined by the TUNEL assay.
(TIF)

Table S1 **Significantly regulated genes.**
(XLS)

Table S2 **Genes in overrepresented biological categories.**
(XLS)

Table S3 **Genes involved in calcium homeostasis (FDR <0.05).**
(XLS)

Method S1 **Microarray Data Analysis.**
(DOC)
