# Supplementary figures and images for: Uropathogenic E. coli Induce Different Immune Response in Testicular and Peritoneal Macrophages: Implications for Testicular Immune Privilege
Source: PLoS One. 2011 Dec 2;6(12):e28452. doi: 10.1371/journal.pone.0028452 (PMC3229579; doi:10.1371/journal.pone.0028452)

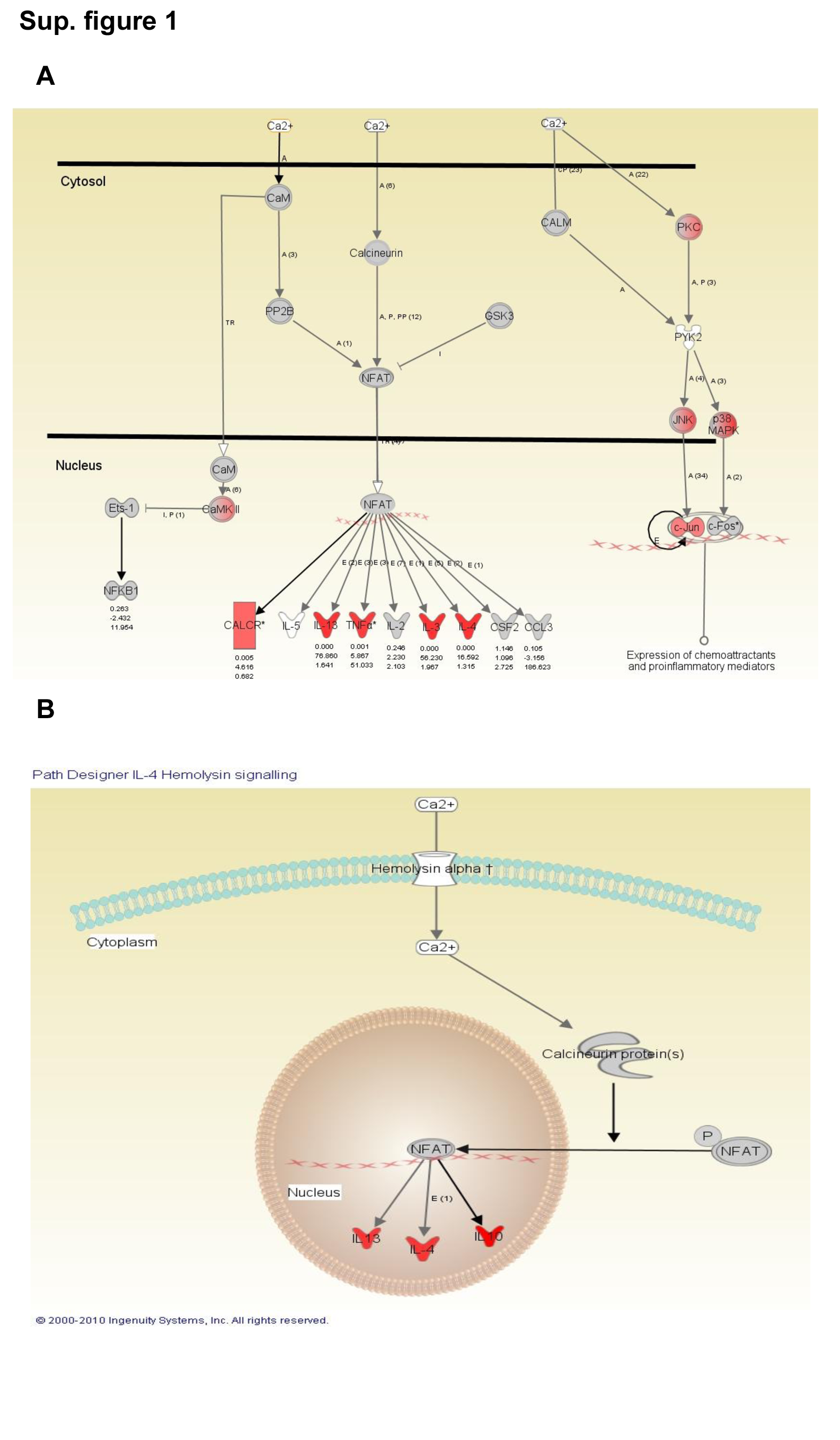

Supplement: Figure S1 — Calcium signaling pathway. (A) Significantly regulated genes in PM and TM were used to generate calcium signaling pathways and identify genes within these pathways using Ingenuity Pathways Analysis (Ingenuity® Systems). Intracellular calcium signaling is mediated in PM 60 min after UPEC infection via calcineurin and nuclear translocation of NFAT which finally leads to the expression of inflammatory cytokines. Rises in intracellular calcium levels also activates protein kinase c (PKC) which in turn activates kinases such as MAPK and JNK. Up regulated genes are depicted in red. B) The picture demonstrates how UPEC alpha hemolysin (HlyA) increases intracellular calcium concentrations which then leads to translocation of NFAT after dephosphorylation by calcineurin. (TIF) [file pone.0028452.s001.tif]

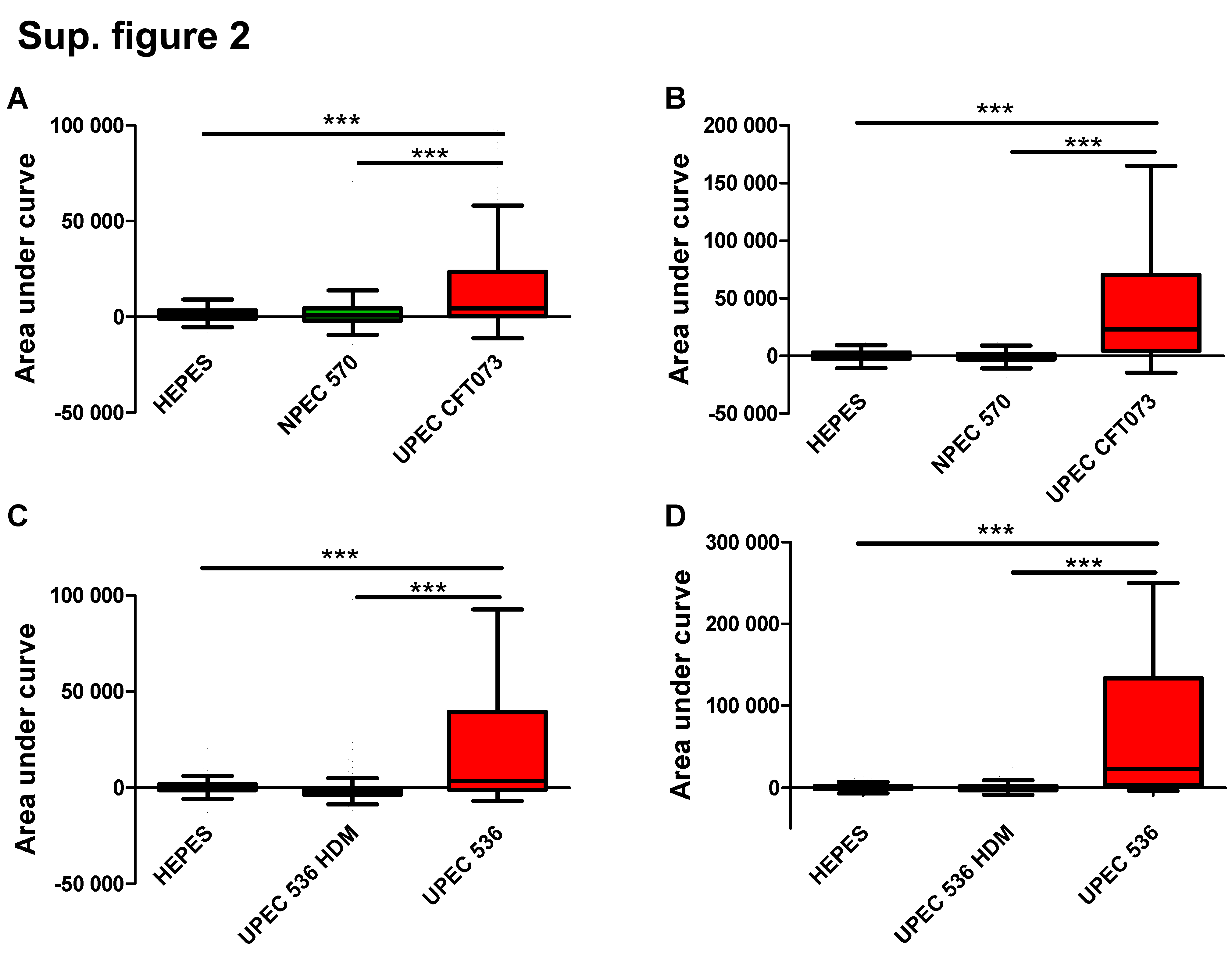

Supplement: Figure S2 — Area under curve analysis for [Ca2+]I data set in Figure 3 . Data were calculated by summing up values obtained for each cell. Rise in [Ca2+]I is caused in (A, C) TM and (B, D) PM by UPEC CFT073 and 536 strain, but not by NPEC 570 and UPEC 536 HDM which lack HlyA. A stronger Ca2+ influx in PM is clearly visible. Non-parametric rank based Kruskal-Wallis test was used to compare multiple groups and if significant differences were detected, it was followed by Mann-Whitney test to compare between two experimental groups. (TIF) [file pone.0028452.s002.tif]

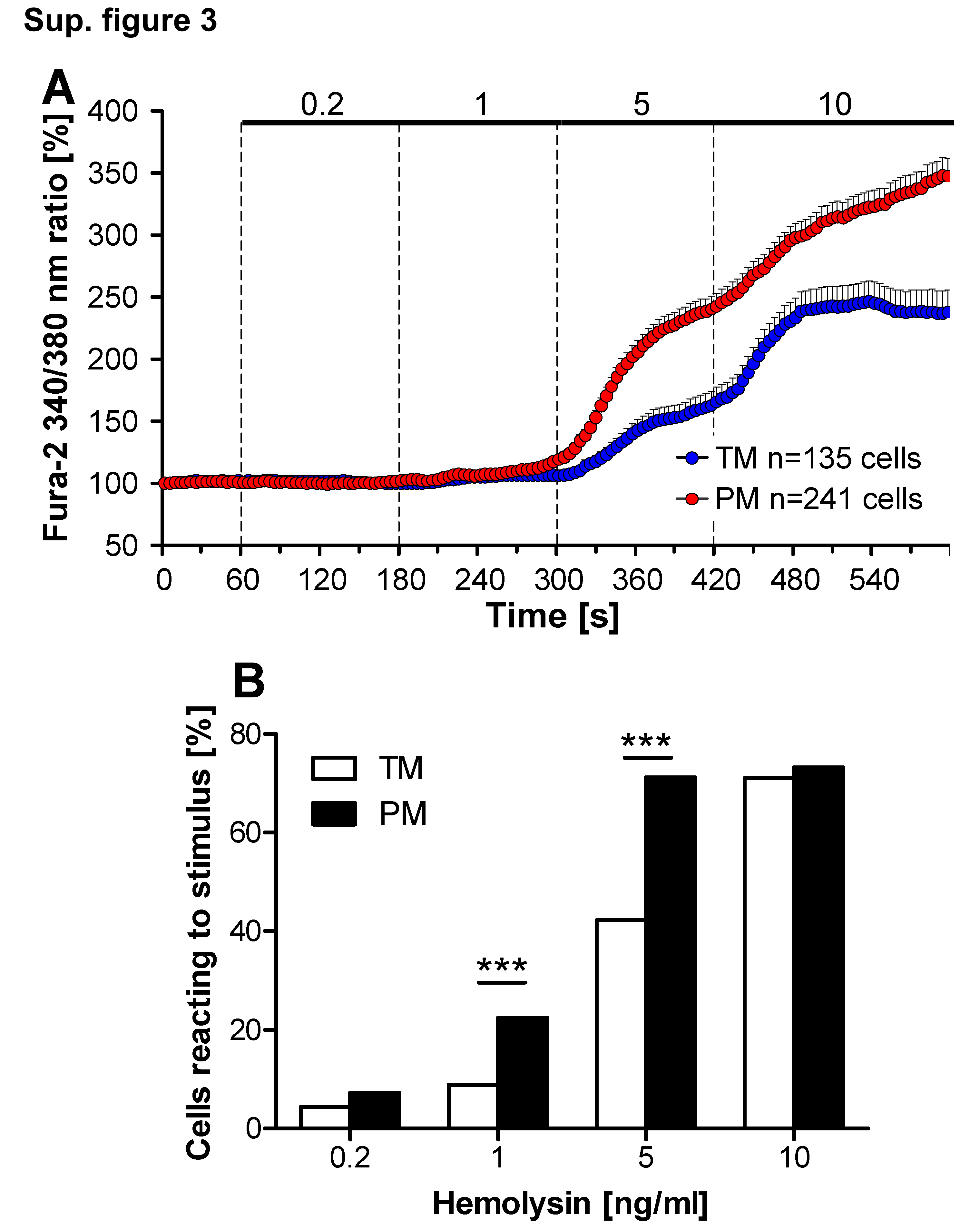

Supplement: Figure S3 — Effect of hemolysin challenge on Ca2+ influx in TM and PM. (A) Cumulative dose-response curve. (B) Number of cells reacting to increasing concentrations of hemolysin A is shown. *** - P≤0.001; differences in numbers of cells reacting to increasing concentrations of HlyA were measured by Fischer’s exact test. (TIF) [file pone.0028452.s003.tif]

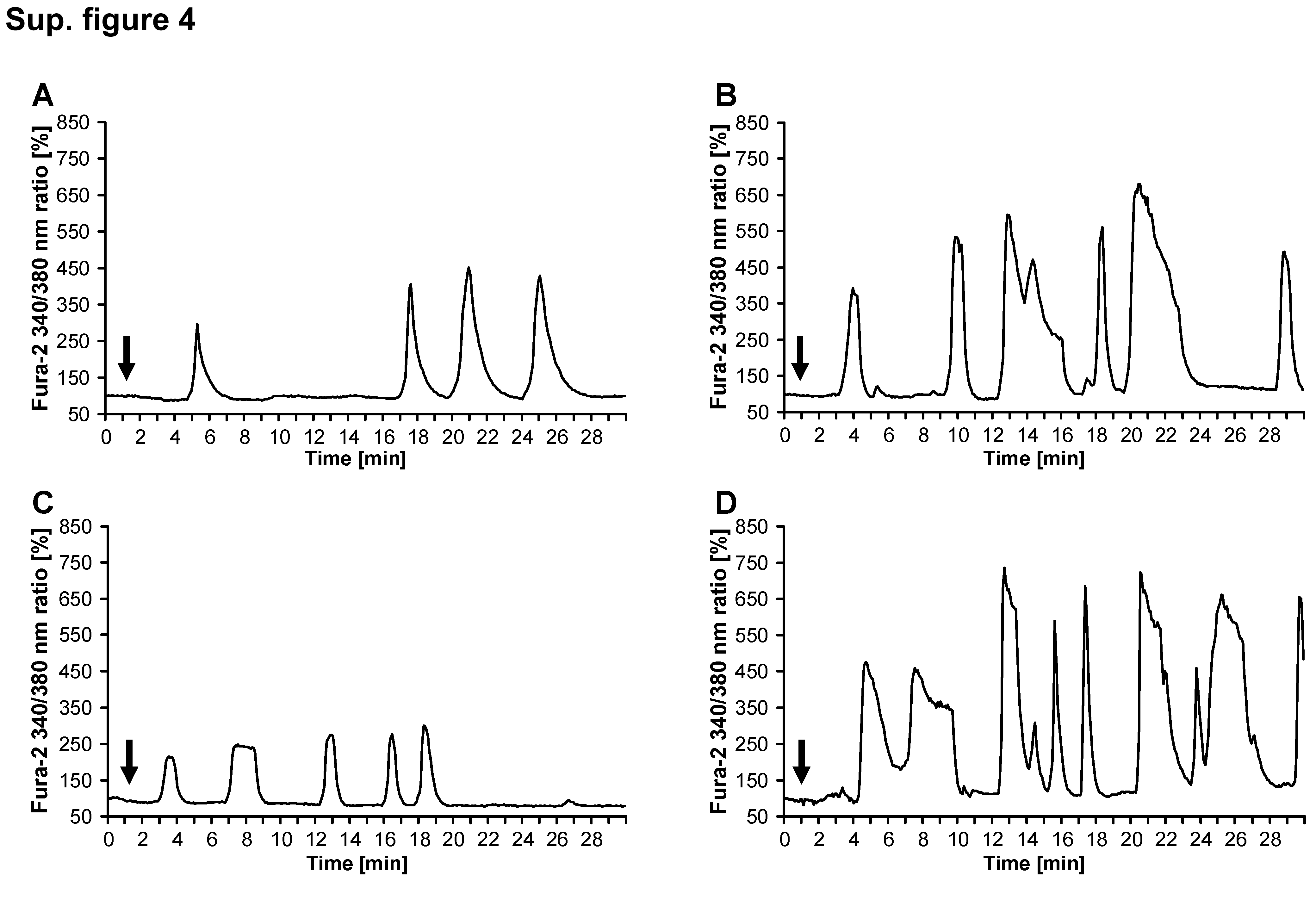

Supplement: Figure S4 — Alpha-hemolysin caused intracellular Ca2+ oscillations in testicular macrophages (TM). Cells were treated with purified alpha-hemolysin (5 ng/ml, as indicated by arrows) and [Ca2+]i was monitored for 30 min using the Fura-2 method. (TIF) [file pone.0028452.s004.tif]

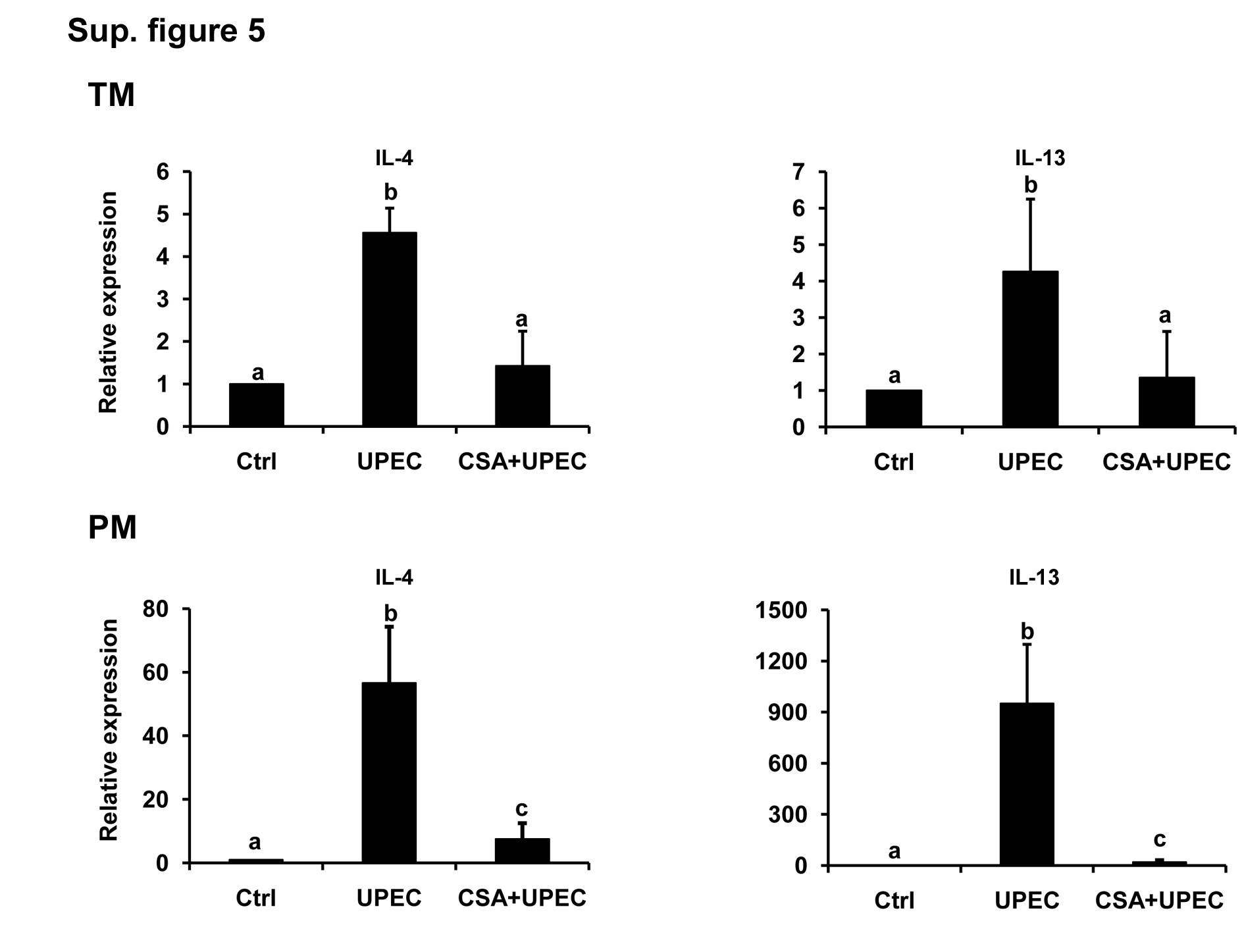

Supplement: Figure S5 — NFAT pathway inhibitor cyclosporine suppressed UPEC induced NFATC2 dependent expression of anti-inflammatory cytokine IL-4 and IL-13. TM and PM were pretreated with 2 µM cyclosporine A (CSA) for 15 min prior challenge with UPEC (MOI = 20) for 1 h. Expression levels of IL-4 and IL-13 were analyzed using qRT-PCR. Results were normalized using β-microglobulin as endogenous controls and are shown as fold changes relative to uninfected controls. Values are means ± SD of triplicates. Mann-Whitney U test was used to analyze data. Values with different letters superscript differ significantly compared to control (Ctrl). (TIF) [file pone.0028452.s005.tif]

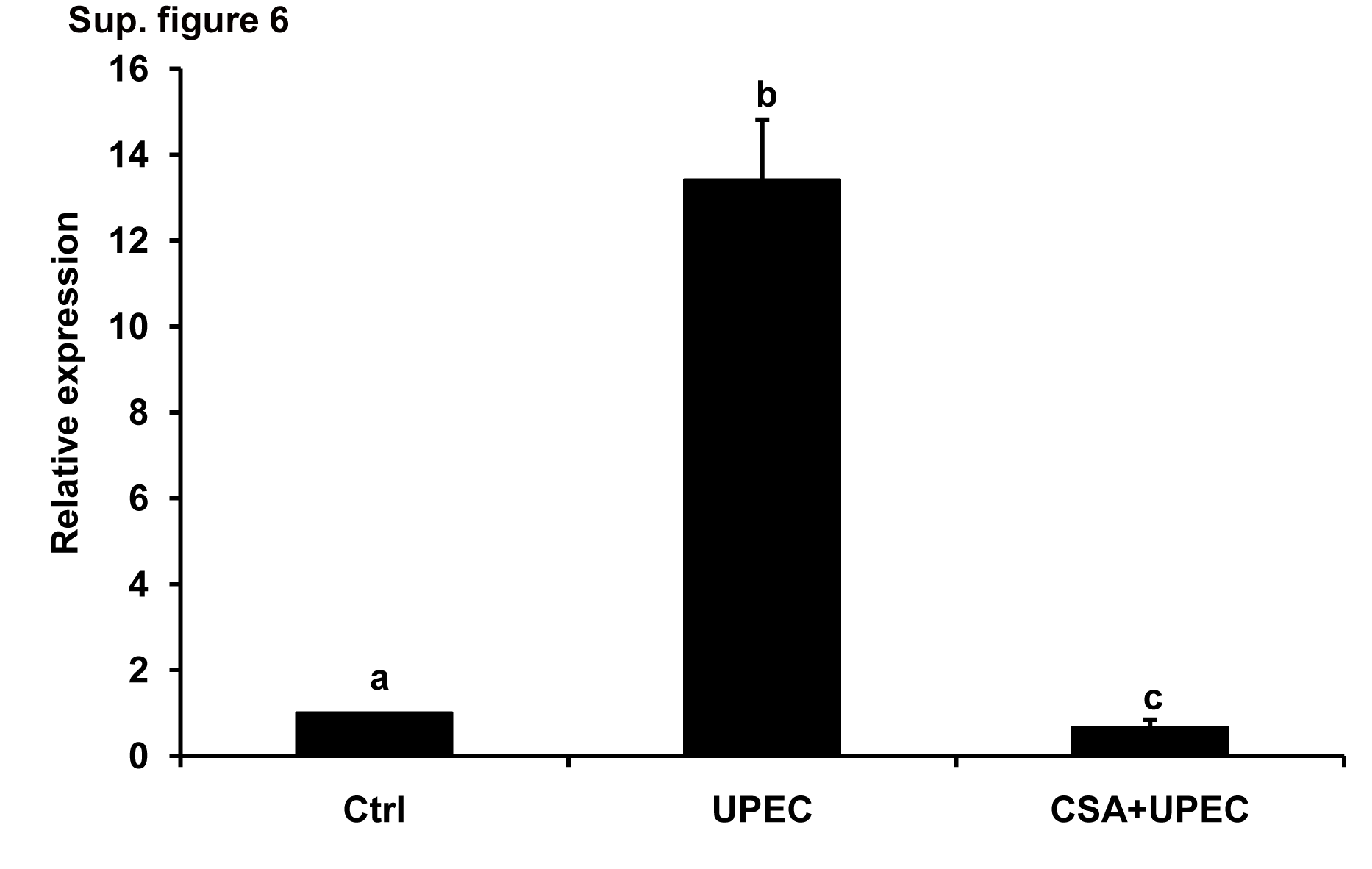

Supplement: Figure S6 — NFAT pathway inhibitor cyclosporine suppressed UPEC induced NFATC2 dependent expression of TNF-α in PM. PM were pretreated with 2 µM cyclosporine A (CSA) for 15 min prior challenge with UPEC (MOI = 20) for 1 h. Expression levels of TNF-α were analyzed using qRT-PCR. Results were normalized using β-microglobulin as endogenous controls and are shown as fold changes relative to uninfected controls. Values are means ± SD of triplicates. Mann-Whitney U test was used to analyze data. Values with different letters superscript differ significantly compared to control (Ctrl). (TIF) [file pone.0028452.s006.tif]

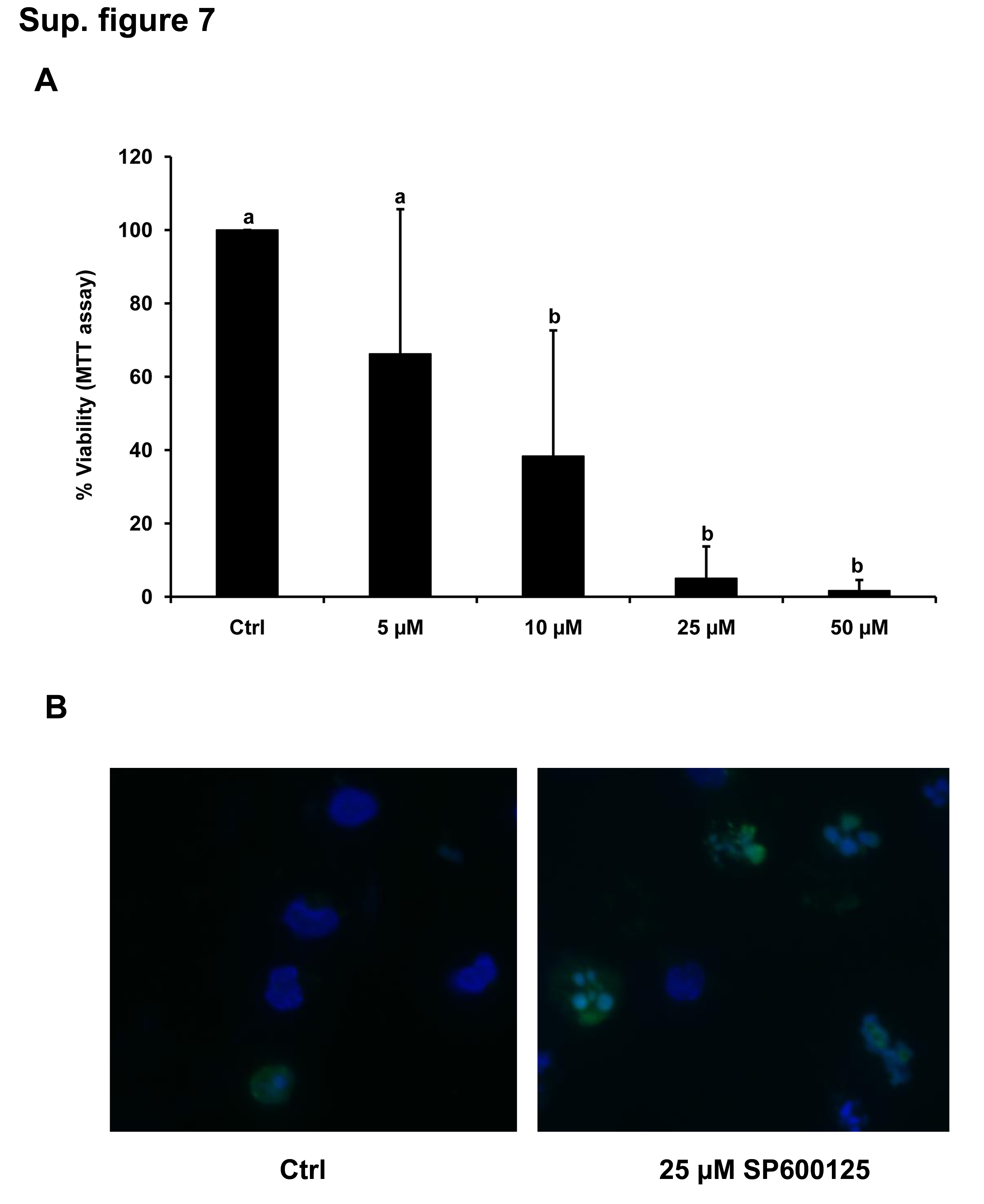

Supplement: Figure S7 — The MAP kinase JNK inhibitor SP600125 induces cell death in PM. (A) PM were treated with JNK inhibitor SP600125 at the indicated concentration for 24 h. Cell viability was determined using the colorimetric MTT assay. Results are presented as means ± SD of triplicates. Mann-Whitney U test was used to analyze data. Values with different letters superscript differ significantly compared to control (Ctrl). (B) PM were treated with 25 µM SP600125 JNK inhibitor for 24 h and DNA fragmentation was examined by the TUNEL assay. (TIF) [file pone.0028452.s007.tif]
