# Supplementary material for: Uropathogenic E. coli Induce Different Immune Response in Testicular and Peritoneal Macrophages: Implications for Testicular Immune Privilege
Source: PLoS One. 2011 Dec 2;6(12):e28452. doi: 10.1371/journal.pone.0028452 (PMC3229579; doi:10.1371/journal.pone.0028452)
Supplement: Method S1 — Microarray Data Analysis. (DOC) [file pone.0028452.s011.doc]

**Microarray Data Analysis**

Spot signals of CodeLink bioarrays were quantified using CodeLink Expression Analysis v4.1, Version 4.1.0.29054 (GE Healthcare) as outlined in the user's manual. CodeLink Expression Software 1.21 generated background corrected raw as well as median centered intra-slide normalized data. The intra-slide normalized data were used for further analysis. The software automatically calculated thresholds for intra-slide normalized intensities for each array and flagged genes as TRUE when the gene intensity was higher than the threshold or FALSE when the intensity was lower. The present call of a microarray was given as the ratio of genes flagged as TRUE / total number of genes on microarray. Microarrays subjected to data analysis showed a mean present call of 84% indicating a high number of genes above threshold, i.e. being flagged as TRUE. Furthermore, the software flagged each gene value as GOOD, EMPTY, POOR, NEG or MSR defining different quality measures as outlined in the user's manual. Only gene values flagged as GOOD or EMPTY were used in the following analysis workflow: 1) Removal of genes with a high number of missing values or of values being flagged as FALSE: genes with missing values >= 50% of all arrays in a group were excluded from the dataset. Genes that were flagged as FALSE in > 50% of arrays in each group were also excluded from the dataset. A total of 32.213 probe sets remained after quality control. 2) Gene expression values deviating more than 3-times from the group median were classified as outliers and have been removed. 3) Imputation of remaining missing values: remaining missing values were imputed using sequential K-nearest neighbor (SKNN) imputation with k=5. 4) Normalization of imputed dataset: imputed dataset was normalized using quantiles normalization in R and logged to base 2. 5) Array outlier detection: dissimilarity matrices of the normalized dataset were generated in AVADIS-Pride to determine outlier arrays within the dataset. No outlier arrays were identified in the data set. 6) Statistical analysis of microarrays: for each gene, the mean value of all technical replicates of an infant was calculated in dChip [35]. To identify differentially regulated genes between different groups, e.g. group 1A versus group 1B, the dataset was subjected to a novel two-class rank statistics (Rank products, RP) as described below . For each gene, a false discovery rate (FDR) < 0.05 was defined as the significance level. 7) Annotation of genes: significantly regulated genes were annotated using the web based annotation tools SOURCE and the Database for Annotation, Visualization and Integrated Discovery (DAVID Bioinformatics Resources 2008) as described in the manual. 9) Enriched functional categories: enriched functional categories within the differentially regulated genes were determined using DAVID version 2.0. DAVID is a platform that provides statistical methods (reported as an Enrichment Score) to facilitate the biological interpretation of gene lists deriving from microarray analysis. Enriched genes describes a class of genes that have similar functions regardless of their expression level, and appear more often in a list of interest than would normally be predicted by their distribution among all genes assayed. An Enrichment Score given as a p-value is calculated for the likelihood of the enrichment of biological processes using the Gene Ontology (GO) public database. 10) Cluster analysis: hierarchical cluster analysis of the significant over- and under-expressed genes was performed using the centroid linkage method and the distance matrix 1 –r in dChip . 10) Pathway analysis: Identification of pathways regulated by the differentially regulated genes was achieved using Pathway Explorer and Ingenuity Pathways Analysis (Ingenuity® Systems, www.ingenuity.com). Canonical pathways analysis identified the pathways from the Ingenuity Pathways Analysis library of canonical pathways that were most significant to the data set. Genes from the data set that met the criteria FDR< 0.05 were associated with a canonical pathway in the Ingenuity Pathways Knowledge Base. The significance of the association between the data set and the canonical pathway was measured in two ways: 1) A ratio of the number of genes from the data set that map to the pathway divided by the total number of genes that map to the canonical pathway. 2) Fischer’s exact test was used to calculate a p-value determining the probability that the association between the genes in the dataset and the canonical pathway is explained by chance alone.

References

1. Kim KY, Kim BJ, Yi GS (2004) Reuse of imputed data in microarray analysis increases imputation efficiency. BMC Bioinformatics 5: 160.

2. Bolstad BM, Irizarry RA, Astrand M, Speed TP (2003) A comparison of normalization methods for high density oligonucleotide array data based on variance and bias. Bioinformatics 19: 185-193.

3. Gwadry FG, Sequeira A, Hoke G, Ffrench-Mullen JM, Turecki G (2005) Molecular characterization of suicide by microarray analysis. Am J Med Genet C Semin Med Genet 133C: 48-56.

4. Breitling R, Armengaud P, Amtmann A, Herzyk P (2004) Rank products: a simple, yet powerful, new method to detect differentially regulated genes in replicated microarray experiments. FEBS Lett 573: 83-92.

5. Breitling R, Herzyk P (2005) Rank-based methods as a non-parametric alternative of the T-statistic for the analysis of biological microarray data. J Bioinform Comput Biol 3: 1171-1189.

6. Diehn M, Sherlock G, Binkley G, Jin H, Matese JC, et al. (2003) SOURCE: a unified genomic resource of functional annotations, ontologies, and gene expression data. Nucleic Acids Res 31: 219-223.

7. Dennis G, Jr., Sherman BT, Hosack DA, Yang J, Gao W, et al. (2003) DAVID: Database for Annotation, Visualization, and Integrated Discovery. Genome Biol 4: P3.

8. Li C, Wong WH (2001) Model-based analysis of oligonucleotide arrays: expression index computation and outlier detection. Proc Natl Acad Sci U S A 98: 31-36.

9. Mlecnik B, Scheideler M, Hackl H, Hartler J, Sanchez-Cabo F, et al. (2005) PathwayExplorer: web service for visualizing high-throughput expression data on biological pathways. Nucleic Acids Res 33: W633-637.
